# Automated sulfur-[^18^F]fluoride exchange radiolabelling of a prostate specific membrane antigen (PSMA) targeted ligand using the GE FASTlab™ cassette-based platform†

**DOI:** 10.1039/d3re00307h

**Published:** 2023-08-21

**Authors:** Zixuan Yang, Chris Barnes, Juozas Domarkas, Joanna Koch-Paszkowski, John Wright, Ala Amgheib, Isaline Renard, Ruisi Fu, Stephen Archibald, Eric O. Aboagye, Louis Allott

**Affiliations:** a Centre for Biomedicine and Positron Emission Tomography Research Centre, Hull York Medical School and University of Hull Cottingham Road Hull HU6 7RX UK louis.allott@hull.ac.uk; b Comprehensive Cancer imaging Centre, Faculty of Medicine, Department of Surgery and Cancer, Imperial College London Hammersmith Hospital, Du Cane Road London UK eric.aboagye@imperial.ac.uk

## Abstract

Sulfur-[^18^F]fluoride exchange radiochemistry is a rapid and convenient method for incorporating fluorine-18 into biologically active molecules. We report a fully automated radiolabelling procedure for the synthesis of a [^18^F]SO_3_F-bearing prostate specific membrane antigen (PSMA) targeted ligand ([^18^F]5) using the GE FASTLab™ cassette-based platform in a 25.0 ± 2.6% radiochemical yield (decay corrected). Uptake *in vitro* and *in vivo* correlated with PSMA expression, and the radioligand exhibited favourable biodistribution and pharmacokinetic profiles.

## Abstract 2

The sulfur-[^18^F]fluoride exchange reaction is a facile ^19^F/^18^F isotopic exchange labelling chemistry which is simple to automated for the preparation of positron emission tomography (PET) radiopharmaceuticals.

## Graphical abstract

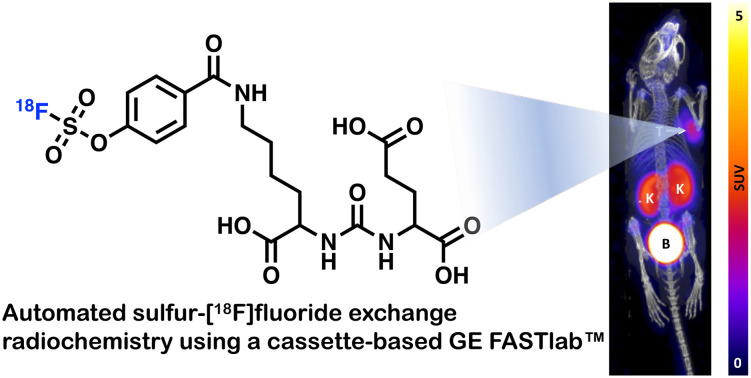

Fluorine-18 (^18^F) is an ideal positron emission tomography (PET) isotope owing to its favourable decay characteristics (*t*_1/2_ = 110 min, β^+^_em_ 0.635 MeV, 97%) and therefore the development of convenient reactions to incorporate the isotope into molecules of interest is an active area of research.^1,2^ The facile sulfur-[^18^F]fluoride exchange reaction is a recent advancement in ^18^F radiochemistry where aryl [^18^F]fluorosulfates are synthesised *via* a ^19^F/^18^F isotopic exchange reaction in high yields, with excellent substate tolerability (Fig. 1).^3^ A variety of ^18^F-labelled molecules have been synthesised using this chemistry, but to our knowledge, an automated cassette-based procedure has not yet been described.^4^ Automation is a key component to the translation of PET radiopharmaceuticals into clinical studies as it allows: 1) scalability of dose while maintaining a safe working environment for production personnel through convenient shielding of the automated system; 2) good manufacturing practice (GMP) compliance to produce safe radiopharmaceutical doses for patients; 3) batch reproducibility and a reduction in batch failure rates.^5,6^ Cassette-based platforms like the GE HealthCare FASTLab™ and FASTLab2™ are a popular choice for radiopharmaceutical manufacturing and are installed in production facilities worldwide. Cassettes are convenient for investigational medicinal product (IMP) and commercial manufacture of radiopharmaceuticals as they can be populated with reagents and essential components in a sterile GMP environment and distributed, allowing for simple “plug and go” synthesis with no requirements for additional radiochemistry development.^5,7^ We have previously demonstrated the flexibility of these platforms in the synthesis of complex radiopharmaceuticals including labelled biologics, some of which have progressed into clinical studies.^8–12^

**Fig. 1:**
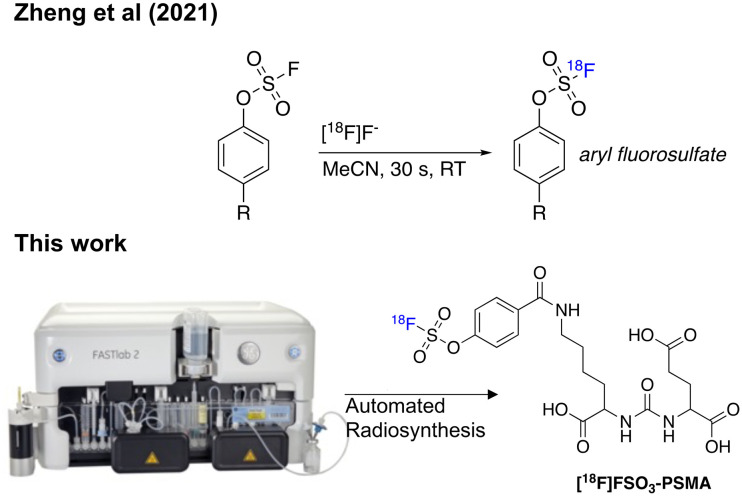
Sulfur-[^18^F]fluoride exchange radiochemistry developed by Zheng *et al.* (2021) and an overview of this study which aimed to automate the radiochemistry using a cassette-based platform.

Herein we report a fully automated procedure for sulfur-[^18^F]fluoride radiochemistry using a commercially available cassette-based platform (GE FASTLab™), exemplified by labelling a prostate specific membrane antigen (PSMA) targeted ligand bearing an aryl fluorosulfates (SO_3_F) moiety.

PET imaging of PSMA expression is a crucial biomarker in metastatic castrate resistant prostate cancer and a variety of radiopharmaceuticals for imaging and molecular radiotherapy have been developed.^13^ The Lys-urea-Glu motif is a simple ligand, which effectively targets PSMA and has been utilised in a variety of applications from radiopharmaceutical development to drug delivery.^14,15^ A SO_3_F-bearing Lys-urea-Glu dipeptide was synthesised following Scheme 1. In brief, H-Lys(Z)-OtBu hydrochloride was reacted with 4-nitrophenyl chloroformate in an inert atmosphere followed by the addition of H-Glu(OtBu)-OtBu hydrochloride to form 1 in a 55% yield after preparative HPLC purification.

**Scheme 1:**
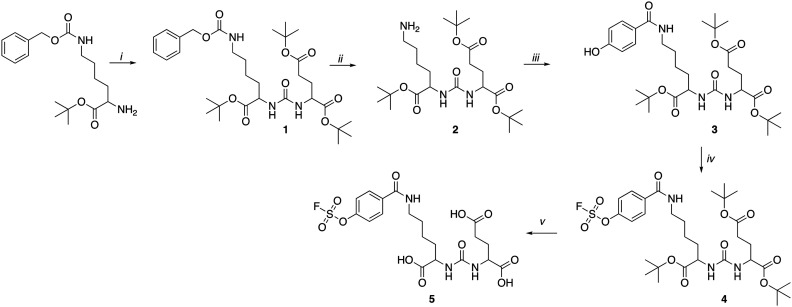
Synthesis of the radiochemistry precursor (4) and reference compound (5). Reaction conditions: i) H-Glu(OtBu)-OtBu. HCl, 4-nitrophenyl chloroformate, DIPEA, anhydrous DCM, 0 °C followed by RT 1 h; ii) H_2_, Pd/C (10%), MeOH, RT, 16 h; iii) 4-hydroxybenzoic acid, HATU, DIPEA, THF, RT, 16 h; iv) AISF, DBU, THF, RT, 1 h; v) TFA, DCM, RT, 16 h.

Hydrogenation of 1 over palladium on carbon (10%) quantitatively liberated the free base 2 which was reacted with commercially available 4-hydroxybenzoic acid to produce 3. The SO_3_F moiety was incorporated into 4 using commercially available 4-[(acetylamino)phenyl]imidodisulfuryl difluoride (AISF) before the hydrolysis of the t-butyl protecting groups in acidic conditions to give the final radiochemistry reference material 5 (SO_3_F-PSMA). All compounds were characterised by MS, ^1^H-NMR, ^13^C-NMR and ^19^F-NMR where appropriate (ESI† Fig. S1–S17). A fluorosulfonyl (SO_2_F) derivative was also synthesised for comparison (ESI,† section 2).

An automated radiosynthesis method to produce [^18^F]SO_3_F-PSMA (**[**^**18**^**F]5**) was developed using the GE FASTLab™ cassette-based platform (Fig. 2). The radiochemistry was performed in two steps within 70 min (Scheme 2). In brief, aqueous [^18^F]fluoride was dried in the presence of K_2_CO_3_ and Kryptofix_222_™ to which precursor 3 (5 μmol) in acetonitrile was added. The reaction proceeded at room temperature for 5 min, before hydrolysis of the *t*-butyl protecting groups in acidic conditions. The crude mixture was purified by semi-preparative HPLC and the desired radioligand was isolated, concentrated and reformulated in ethanol using a tC18 plus SPE cartridge. The radiochemical yield (RCY) non-decay corrected was 25.0 ± 2.6% (*n* = 3), and the molar activity was 0.13 ± 0.08 GBq μmol^−1^ (*n* = 3) from low starting activities of 1.75 ± 0.5 GBq (*n* = 3). Generally, the molar activities of radioconjugates labelled *via*^19^F/^18^F isotopic exchange are notoriously low as unlabelled precursor cannot be isolated from labelled product; however, the molar activities reported here were sufficient for biological evaluation, even when starting from low levels of [^18^F]fluoride. The use of larger activities of [^18^F]fluoride would undoubtedly lead to higher molar activity products, and could be investigated in the future, but was not necessary for this study. The identity and radiochemical purity of **[**^**18**^**F]5** was determined by HPLC (Scheme 2B). The stability of **[**^**18**^**F]5** in formulation was >99% over the duration of testing (4 hours). Given the simplicity of the radiochemistry and the previously exemplified tolerability of sulfur-[^18^F]fluoride exchange reactions towards an extensive scope of substrates, it is anticipated that this automated procedure could be adapted to label other ligands, though substrate specific optimisation such as precursor quantity and purification strategy will be required. An SO_2_F bearing PSMA conjugate was also successfully radiolabelled using this method (ESI†). To our knowledge, this is the first time that an SO_2_F moiety has been labelled *via* isotopic exchange as literature examples displace a chloro-leaving group (SO_2_Cl).^16^

**Fig. 2:**
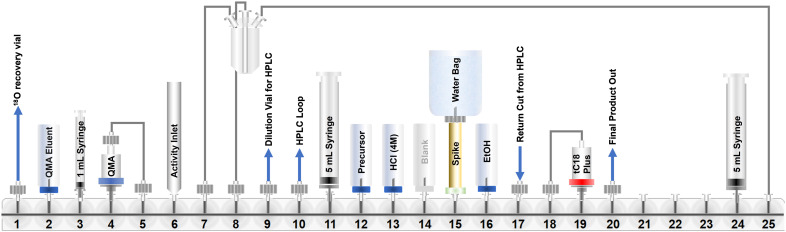
A schematic representation of the GE FASTLab™ cassette developed for the automated radiosynthesis of **[**^**18**^**F]5**. Full details of the cassette setup and radiochemistry methodology are presented in the ESI† (section 3).

**Scheme 2:**
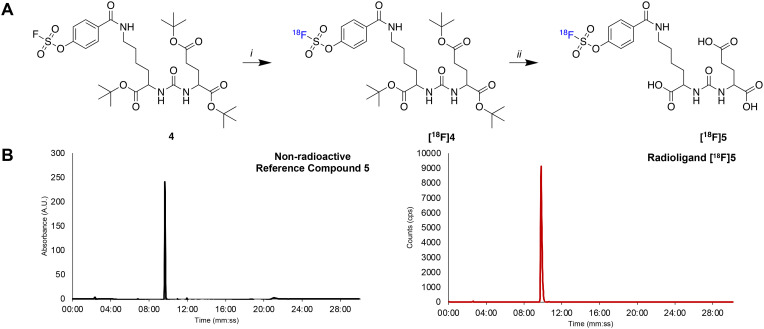
A) Radiosynthesis of **[**^**18**^**F]5**. Reaction conditions: i) K_222_–K[^18^F]F, MeCN, RT, 5 min; ii) HCl (4 M) 60 °C, 15 min; B) corresponding HPLC chromatograms showing non-radioactive reference standard 5 (*t*_R_ = 09:39 mm:ss) and **[**^**18**^**F]5** (*t*_R_ = 09:48 mm:ss).

Evaluation of **[**^**18**^**F]5** was performed *in vitro* and *in vivo* in PSMA expressing models to confirm the targeting properties of the radioligand. The uptake of **[**^**18**^**F]5** was evaluated in a panel of cell lines with differential expression of PSMA (ESI† Fig. S20); PSMA expression was confirmed in the cell lines by flow cytometry (ESI† Fig. S19). While cellular uptake of **[**^**18**^**F]5** was generally low (0.05–0.5% ID g^−1^), a clear and statistically significant trend was observed where uptake of **[**^**18**^**F]5** was highest in PSMA positive cells. Uptake in LNCaP and C4-2B cells was 7.7 and 8.7-fold higher, respectively, compared to PSMA negative prostate cancer cells PC3, and normal cells PNT1A. These data confirm that **[**^**18**^**F]5** retained PSMA-target recognition which encouraged *in vivo* studies.

The biodistribution of **[**^**18**^**F]5** was first evaluated by *ex vivo* gamma counting of tissue (Fig. 3A) in naïve mice. Renal elimination was the primary route clearance (2.2 ± 2.0% ID g^−1^), which is characteristic for hydrophilic peptides. Uptake in muscle was low (0.1 ± 0.4% ID g^−1^), ideal for high contrast PET images in tumour bearing animals. PET imaging in naïve mice showed low skeletal uptake, which was indicative of *in vivo* stability towards defluorination (ESI† Fig. S21). Radioactive metabolite analysis highlighted that **[**^**18**^**F]5** exhibited excellent stability in key tissues and organs (Fig. 3C). An [^18^F]FSO_2_-PSMA ligand was also evaluated *in vivo* and extensive defluorination was observed, which was in agreement with *in vitro* metabolite studies reported in the literature (ESI† Fig. S26).^16^

**Fig. 3:**
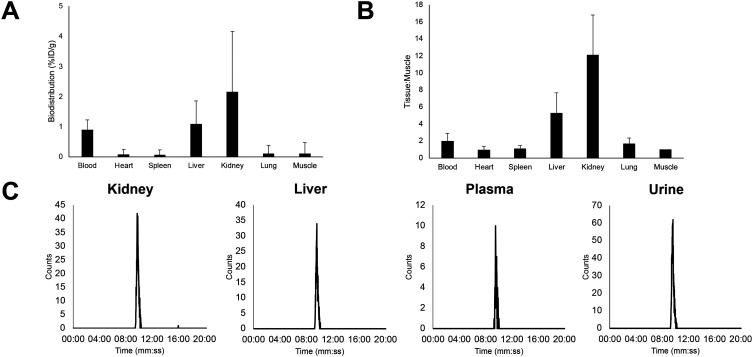
*Ex vivo* biodistribution of **[**^**18**^**F]5** in tumour-naïve mice (*n* = 4) with A) showing uptake (% ID g^−1^) in key organs and B) tissue : muscle ratio; C) representative radioactive metabolite analysis HPLC chromatograms at 60 min p.i. (*n* = 2).

Compound **[**^**18**^**F]5** was then evaluated *in vivo* using LNCaP tumour bearing mice (*n* = 3). Dynamic PET imaging over 90 min showed significant tumour uptake and tumour : muscle contrast (Fig. 4). Time activity curves showed consistent retention of radioactivity in the tumour after *ca.* 30 min including significant and sustained tumour : muscle contrast (5.6 ± 2.7) even at 90 min post-injection (Fig. 4C). All PET images and TACs are presented in the ESI† (Fig. S22–S24).

**Fig. 4:**
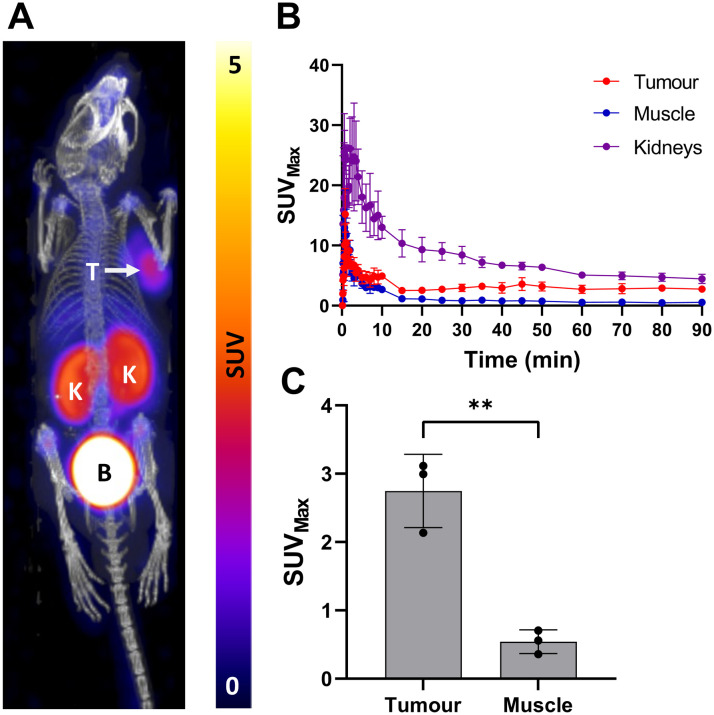
*In vivo* evaluation of **[**^**18**^**F]5** in male BALB/c nude mice bearing LNCaP tumours (*n* = 3); A) dynamic PET/CT image of summed frames from 80–90 min. K = kidney, B = bladder, T = LNCaP tumour; B) time activity curves generated from drawn regions of interest (ROIs) over 90 min PET/CT scan; C) a bar graph showing tumour and muscle uptake (SUV_Max_) at 90 min. Data represented as mean ± SD (*n* = 3, **P = 0.0025).

Sulfur-[^18^F]fluoride exchange radiochemistry is a facile technique for labelling biologically relevant molecules, and is a welcome addition to the radiochemistry toolbox of ^18^F-fluorination chemistry. This cassette-based automated radiolabelling method will help progress sulfur-[^18^F]fluoride exchange radiochemistry towards clinical evaluation as GMP grade radiopharmaceuticals. This work has built a platform for others to develop and optimise for their substrates of interest; work to further improve labelling efficiency, evaluate the use of clinically relevent activities of fluorine-18 (>30 GBq), and optimise for clinically relevant molar activities would be required to translate radioligands produced using this protocol into clinical applications. We exemplified the automated method through the synthesis of radioligand **[**^**18**^**F]5**, which not only effectively demonstrated the utility of the platform, but has highlighted a new radioligand with favourable *in vitro* selectivity towards PSMA expressing cell lines, and favourable *in vivo* uptake, metabolic stability, biodistribution and pharmacokinetics profile. Further biological investigation of this radioligand is warranted.

## Conflicts of interest

There are no conflicts to declare.

## Supplementary Material

RE-008-D3RE00307H-s001
