# Supplementary material for: Automated sulfur-[18F]fluoride exchange radiolabelling of a prostate specific membrane antigen (PSMA) targeted ligand using the GE FASTlab™ cassette-based platform
Source: React Chem Eng. 2023 Aug 21;8(10):2403–7. doi: 10.1039/d3re00307h (PMC10520611; doi:10.1039/d3re00307h)
Supplement: RE-008-D3RE00307H-s001 [file RE-008-D3RE00307H-s001.pdf]

Electronic Supplementary Material (ESI) for Reaction Chemistry & Engineering.
This journal is © The Royal Society of Chemistry 2023

Automated sulfur-[^{18}F]fluoride exchange radiolabelling of a prostate specific membrane antigen (PSMA) targeted ligand using the GE FASTlab™ cassette-based platform

Zixuan Yang,[b] Chris Barnes,[b] Juozas Domarkas,[a] Joanna Koch-Paszkowski,[a] John Wright,[a] Ala Amgheib,[b] Isaline Renard,[a] Ruisi Fu,[b] Stephen Archibald,[a] Eric O. Aboagye*[b] and Louis Allott*[a]

[a.] *Positron Emission Tomography Research Centre, Faculty of Health Sciences, University of Hull, Cottingham Road, Kingston upon Hull, UK. *Corresponding author: (louis.allott@hull.ac.uk)*

[b.] *Comprehensive Cancer imaging Centre, Faculty of Medicine, Department of Surgery and Cancer, Imperial College London, Hammersmith Hospital, Du Cane Road, London, UK. *Corresponding author: (eric.aboagye@imperial.ac.uk)*

Electronic Supporting Information

Contents

1.0 Materials and Methods Page 03

2.0 Synthesis Page 04-15

3.0 Radiochemistry Page 15-17

4.0 Biological Evaluation Page 17-24

1.0 Materials and Methods

Anhydrous solvents and reagents were purchased from Sigma Aldrich (Gillingham, UK) and used without additional purification. Flash column chromatography was performed using silica gel (Merck Kieselgel 60 F254 320-400 mesh). The production of [^{18}F]fluoride for use at Imperial College London was performed using a Siemens RDS111 cyclotron by irradiation of an enriched [^{18}O]H_2O target, supplied by Invicro (London, UK). The production of [^{18}F]fluoride for use at the University of Hull was performed using Best ABT Inc. BG-75 cyclotron (7.5 MeV, 5 μA beam, 1 h) with a target containing 98% ^{18}O enriched water (Marshall Isotopes Ltd, Israel). Automated radiosynthesis was performed using the GE FASTlab™ (GE Healthcare Life Sciences, Amersham, UK). Solid phase extraction (SPE) cartridges were purchased from Waters (Hertfordshire, UK) and used according to the manufacturer's guidelines. Semi-preparative reverse phase (RP) HPLC was performed using a Shimadzu LC20-AT pump attached to a custom-built system, equipped with an Phenomenex Luna C12, 4 μ (250 x 9.4 mm) column running an isocractic mobile phase of MeCN (30%) and H_2O (70%) + 0.1% H_3PO_4 at a flow rate of 3 mL/min. Reaction efficiency and radioactive product identity was determined by RP-HPLC using an Agilent 1200 series instrument connected to a flow-ram detector (Lablogic, Sheffield, UK).

2.0 Synthesis

2.1 Synthesis of SO_3F-PSMA

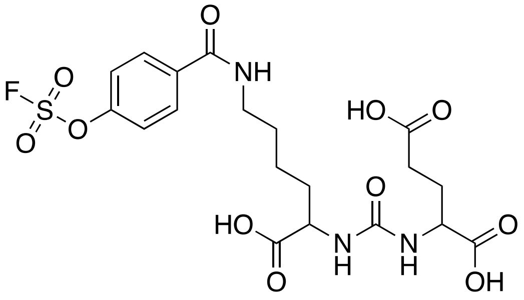

2.1.1 Synthesis of (Bz)Lys-OtBu-N-alpha-uredo-Glu(OtBu)-OtBu (1). H-Glu(OtBu)-OtBu·HCl (10.14 mmol, 3.0 g) and 4-nitrophenyl chloroformate (11.16 mmol, 2.25 g) were dissolved in anhydrous DCM (30 mL) and stirred at 0 °C under an inert atmosphere. Diisopropylethylamine (22.31 mmol, 4.0 mL) was added dropwise to the reaction mixture to maintain 0 °C, after which the reaction was stirred for 5 min and warmed to RT over 30 min. H-Lys(Z)-OtBu·HCl (12.17 mmol, 4.5 g) was added followed by dropwise addition of DIPEA (23.32 mmol, 4.2 mL). The reaction was stirred at RT for 1 h, then evaporated to dryness and purified by silica gel flash chromatography (EtOAc:hexane, 2:1) and preparative HPLC (A: H_2O + 0.1% TFA, B: MeCN. Gradient: 5-95% B, product eluted at 65-70% MeCN). Product was obtained as colorless viscous oil (3.5 g, 55% yield). ^{1}H NMR (400 MHz, Chloroform-*d*) δ 7.37 – 7.26 (m, 5H), 5.43 – 5.26 (m, 3H), 5.17 – 5.00 (m, 2H), 4.38 – 4.25 (m, 2H), 3.16 (hept, *J* = 6.7 Hz, 2H), 2.34 – 2.21 (m, 2H), 2.10 – 1.98 (m, 1H), 1.86 – 1.66 (m, 2H), 1.63 – 1.48 (m, 3H), 1.41 (s, 27H), 1.37 – 1.26 (m, 2H). ^{13}C NMR (101 MHz, Chloroform-*d*) δ 172.61, 172.52, 157.14, 156.76, 128.57, 128.16, 128.10, 82.24, 81.80, 80.62, 66.63, 53.40, 53.06, 40.78, 32.73, 31.70, 29.46, 28.18, 28.12, 28.10, 22.41. MS (ES-ToF) m/z calcd: 621.77; found [M+H]: 622.37.

2.1.2 Synthesis of Lys-OtBu-N-alpha-uredo-Glu(OtBu)-OtBu (2). Compound 1 (5.568 mmol, 3.5 g) was dissolved in MeOH (100 mL) which was evacuated and refilled with N_2 three times. Pd/C (10%, 200 mg) was added and flushed with N_2. The flask was flushed with H_2 via a balloon stirred for 16 h at room temperature. Upon completion, celite (2 g) was added and stirred with for 5 min before filtration and solvent was removed *in vacuo*. Compound 2 was obtained as viscous colorless oil (2.2 g, 81% yield). ^{1}H NMR (400 MHz, Chloroform-*d*) δ 5.37 – 5.25 (m, 2H), 4.37 – 4.27 (m, 2H), 2.40 – 2.23 (m, 2H), 2.06 (dddd, *J* = 14.0, 9.3, 6.5, 4.9 Hz, 1H), 1.89 – 1.71 (m, 2H), 1.68 – 1.57 (m, 1H), 1.54 – 1.47 (m, 2H), 1.46 – 1.40 (m, 27H), 1.38 – 1.23 (m, 2H). ^{13}C NMR (101 MHz, Chloroform-*d*) δ 172.72, 172.61, 172.56, 157.04, 82.22, 81.86, 80.69, 53.50, 53.13, 41.57, 32.84, 32.34, 31.74, 28.45, 28.20, 28.15, 28.13, 22.36. MS (ES-ToF) m/z calcd: 487.64; found [M+H]: 488.33.

2.1.3 Synthesis of di-*tert*-butyl ((1-(*tert*-butoxy)-6-(4-hydroxybenzamido)-1-oxohexan-2-yl)carbamoyl)glutamate (3). 4-hydroxybenzoic acid (0.615 mmol, 84.97 mg), HATU (0.45 mmol, 171.14 mg) and DIPEA (0.82 mmol, 142.84 µL) were dissolved in THF (4 mL), and stirred at RT for 15 min. Compound **2** (0.41 mmol, 200 mg) was added and stirred for 16 h at RT. Then the reaction mixture was evaporated to dryness, followed by extraction with DCM, wash with brine, and purified by silica gel flash chromatography (EtOAc/Hexane=1/1). Compound **3** was obtained as white solid with the yield of 210 mg, 84%.^{1}H NMR (400 MHz, Chloroform-*d*) δ 7.68 – 7.62 (m, 2H), 6.83 – 6.78 (m, 2H), 5.72 (dd, *J* = 16.5, 8.0 Hz, 2H), 4.25 (td, *J* = 8.2, 5.2 Hz, 2H), 3.36 – 3.27 (m, 2H), 2.28 (dt, *J* = 8.7, 6.3 Hz, 2H), 1.85 – 1.66 (m, 2H), 1.53 (q, *J* = 6.7 Hz, 4H), 1.42 – 1.37 (m, 27H), 1.34 – 1.29 (m, 2H). ^{13}C NMR (101 MHz, Chloroform-*d*) δ 172.79, 172.73, 172.68, 168.33, 157.73, 129.14, 125.52, 115.47, 82.34, 81.92, 80.90, 53.55, 53.15, 50.66, 39.83, 31.67, 31.00, 29.00, 28.13, 28.04, 28.01, 22.73, 21.12. MS (ES-ToF) m/z calcd: 607.75; found [M+H]: 608.35.

2.1.4 Synthesis of di-tert-butyl ((1-(tert-butoxy)-6-(4-((fluorosulfonyl)oxy)benza-mido)-1-oxohexan-2-yl)carbamoyl)glutamate (4). Compound **3** (0.099 mmol, 60 mg), AISF (0.197 mmol, 61.9 mg), and DBU (0.148 mmol, 22.2 µL) were dissolved in THF (2 mL) and stirred at RT for 1 h. Solvent was removed under vacuum and the crude purified by silica gel flash chromatography (EtOAc/Hexane=1.5/1). **6** was obtained as white solid with the yield of 65 mg, 95%.^{1}H NMR (400 MHz, Chloroform-*d*) δ 8.12 – 8.08 (m, 2H), 7.40 – 7.37 (m, 2H), 4.21 – 4.10 (m, 3H), 3.54 (dtd, *J* = 11.4, 7.0, 6.5, 3.4 Hz, 1H), 3.38 – 3.29 (m, 1H), 2.27 (dt, *J* = 8.5, 6.4 Hz, 2H), 2.02 – 1.95 (m, 2H), 1.79 – 1.71 (m, 2H), 1.57 – 1.44 (m, 4H), 1.40 (d, *J* = 2.7 Hz, 27H), 1.23 (t, *J* = 7.1 Hz, 2H). ^{13}C NMR (101 MHz, Chloroform-*d*) δ 174.26, 172.41, 172.06, 165.89, 157.71, 151.76, 135.20, 130.18, 120.79, 82.88, 81.66, 80.86, 54.08, 53.22, 40.16, 32.45, 31.56, 29.15, 28.11, 28.06, 27.94, 23.95. ^{19}F NMR (376 MHz, Chloroform-*d*) δ 38.24. MS (ES-ToF) m/z calcd: 689.79; found [M+H]: 690.31.

2.1.5 Synthesis of ((1-carboxy-5-(4-((fluorosulfonyl)oxy)benzamido)pentyl)carb-amoyl)glutamic acid (5). Compound **4** (0.02 mmol, 15 mg) was dissolved in DCM (1 mL), TFA (250 µL) was added to the solution and stirred for 16 h at RT. Solvent was removed *in vacuo* and the crude was directly used as radiochemical reference compound in analytical HPLC without purification. ^{1}H NMR (400 MHz, Methanol-d4) δ 8.02 – 7.95 (m, 2H), 7.59 – 7.52 (m, 2H), 4.30 (ddd, J = 8.4, 6.4, 4.9 Hz, 2H), 3.40 (td, J = 7.0, 2.4 Hz, 2H), 2.47 – 2.36 (m, 2H), 2.15 (dddd, J = 19.3, 8.7, 7.3, 4.8 Hz, 2H), 1.96 – 1.84 (m, 2H), 1.68 (dq, J = 11.8, 5.0, 3.5 Hz, 2H), 1.51 (q, J = 7.6 Hz, 2H). ^{13}C NMR (101 MHz, Methanol-d4) δ 153.27, 136.68, 122.24, 54.79, 53.94, 40.90, 33.24, 31.02, 29.89, 28.82, 24.02. ^{19}F NMR (376 MHz, Methanol-d4) δ -76.87. MS (ES-ToF) m/z calcd: 521.47; found [M+H]: 522.12.

2.2 Synthesis of SO_2F-PSMA

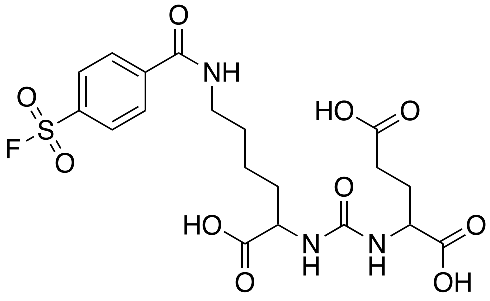

2.2.1 Synthesis of di-*tert*-butyl ((1-(*tert*-butoxy)-6-(4-(fluorosulfonyl)benzamido)-1-oxohexan-2-yl)carbamoyl)glutamate (6). Compound **2** (0.22 mmol, 109 mg) was dissolved in anhydrous DCM (2 mL), and triethylamine (0.02 mmol, 3 µL) was added to the reaction. The reaction mixture was cooled to 0 °C, followed by the addition of 4-fluorosulfonyl benzoic acid chloride (4, 0.21 mmol, 46 mg) to the solution and stirred at 0 °C for 3 h. HCL (1M, 10 mL) was added to the solution and extracted twice with EtOAc (15 mL). The organic layer was washed with sat. $NaHCO_3$ (50 mL), brine (50 mL), and dried over $MgSO_4$. The organic layer was evaporated *in vacuo* and the residue purified by silica gel flash chromatography (EtOAc:hexanes, 1:1). Compound **6** was obtained as white solid (82mg, 55% yield). ^{1}H NMR (400 MHz, Chloroform-*d*) δ 8.22 – 8.17 (m, 2H), 8.09 – 8.05 (m, 2H), 5.86 (s, 1H), 5.53 (s, 1H), 4.23 – 4.11 (m, 2H), 3.57 – 3.34 (m, 2H), 2.38 – 2.20 (m, 2H), 2.06 – 1.96 (m, 1H), 1.78 (dddd, *J* = 16.2, 11.8, 8.2, 5.1 Hz, 2H), 1.71 – 1.48 (m, 3H), 1.37 (s, 27H). ^{13}C NMR (101 MHz, Chloroform-*d*) δ 172.42, 172.14, 165.57, 157.79, 141.31, 128.51, 82.90, 81.88, 80.94, 53.85, 53.21, 40.28, 32.56, 31.51, 28.77, 28.08, 28.03, 27.95, 27.80, 23.63. MS (ES-ToF) m/z calcd: 673.79; found [M+H]: 674.31.

2.2.2 Synthesis of ((1-carboxy-5-(4-((fluorosulfonyl)benzamido)pentyl)carbamoyl)glutamic acid (7). To a solution of **6** (26 mg, 0.039 mmol) dissolved in DCM (1 mL) TFA (250 µL) was added dropwise and stirred overnight under N2 at RT. Reaction mixture was dried and **7** was obtained as white solid. ^{1}H NMR (400 MHz, Methanol-d4) δ 8.12 – 8.07 (m, 2H), 8.04 (d, J = 8.3 Hz, 2H), 4.24 (dt, J = 8.3, 5.1 Hz, 2H), 3.36 (td, J = 7.0, 2.0 Hz, 2H), 2.42 – 2.28 (m, 2H), 2.07 (dtt, J = 14.8, 12.7, 6.2 Hz, 1H), 1.96 – 1.75 (m, 2H), 1.62 (ddt, J = 13.7, 10.6, 7.2 Hz, 3H), 1.53 – 1.35 (m, 2H). ^{13}C NMR (101 MHz, Methanol-d4) δ 176.68, 176.61, 176.07, 168.17, 160.33, 130.13, 129.98, 54.14, 53.70, 41.20, 31.29, 29.99, 29.07, 24.21. MS (ES-ToF) m/z calcd: 505.47; found [M-H]: 504.12.

2.3 NMR Data

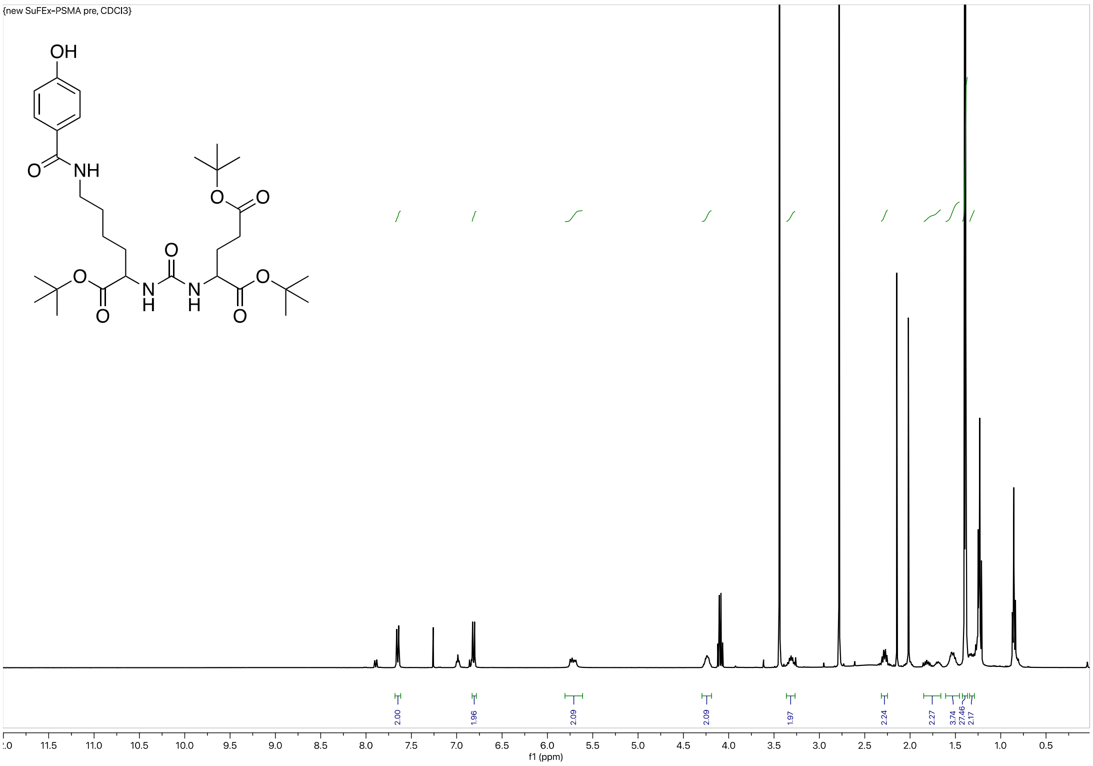

Figure 1. ^{1}H-NMR of di-tert-butyl ((1-(tert-butoxy)-6-(4-hydroxybenzamido)-1-oxohexan-2-yl)carbamoyl)glutamate (**3**).

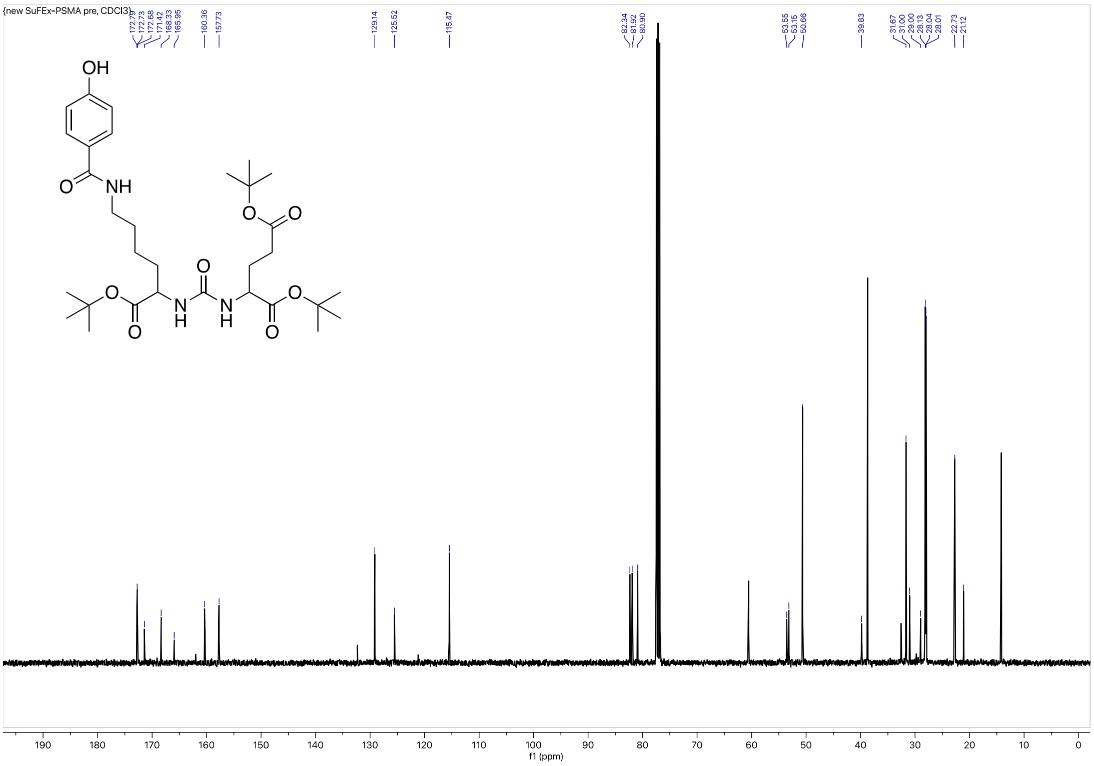

Figure 2. ^{13}C-NMR of di-tert-butyl ((1-(tert-butoxy)-6-(4-hydroxybenzamido)-1-oxohexan-2-yl)carbamoyl)glutamate (**3**).

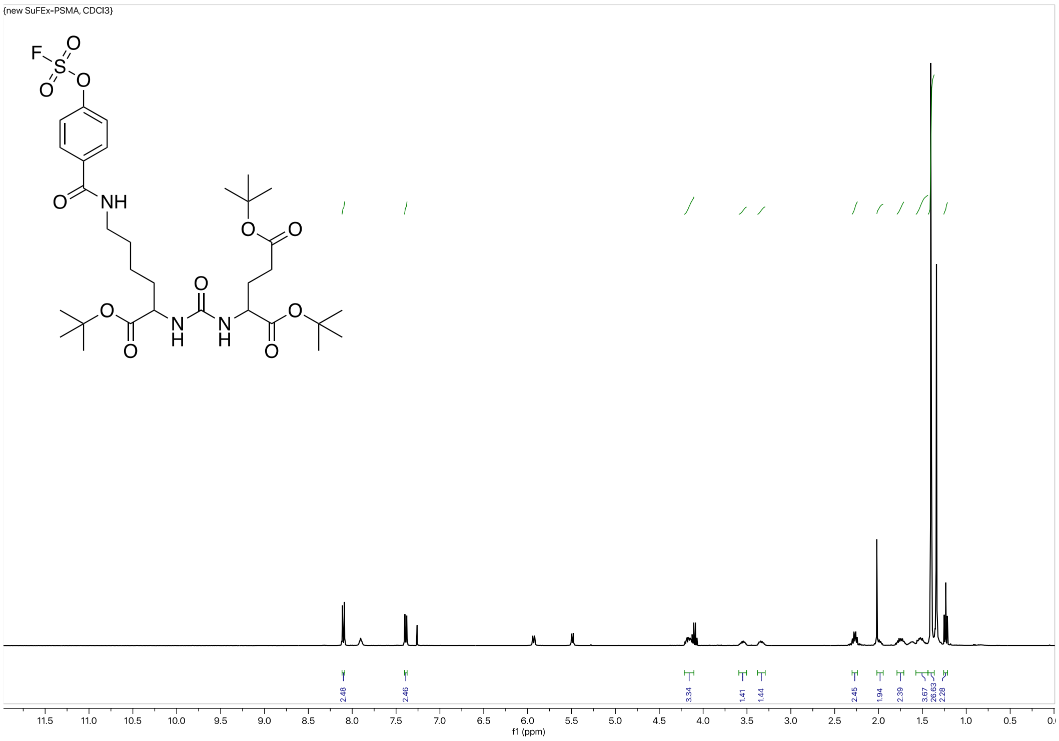

Figure 3. ^{1}H-NMR of di-tert-butyl ((1-(tert-butoxy)-6-(4-((fluorosulfonyl)oxy)benzamido)-1-oxohexan-2-yl)carbamoyl)glutamate (**4**).

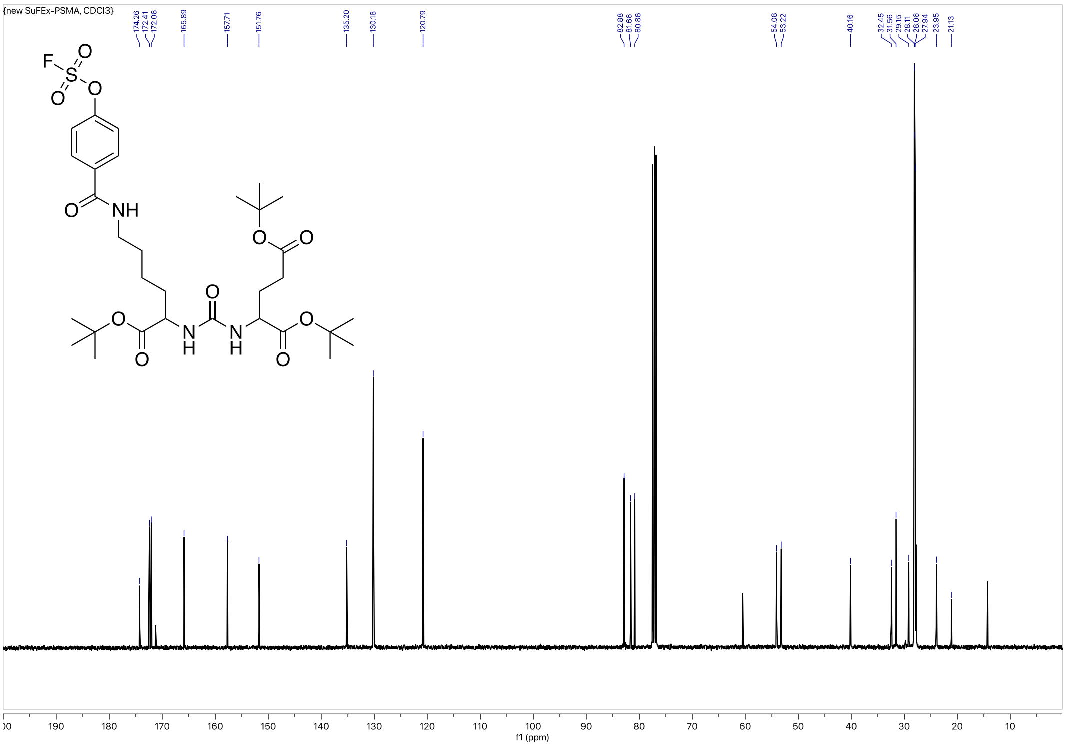

Figure 4. ^{13}C-NMR of di-tert-butyl ((1-(tert-butoxy)-6-(4-((fluorosulfonyl)oxy)benzamido)-1-oxohexan-2-yl)carbamoyl)glutamate (**4**).

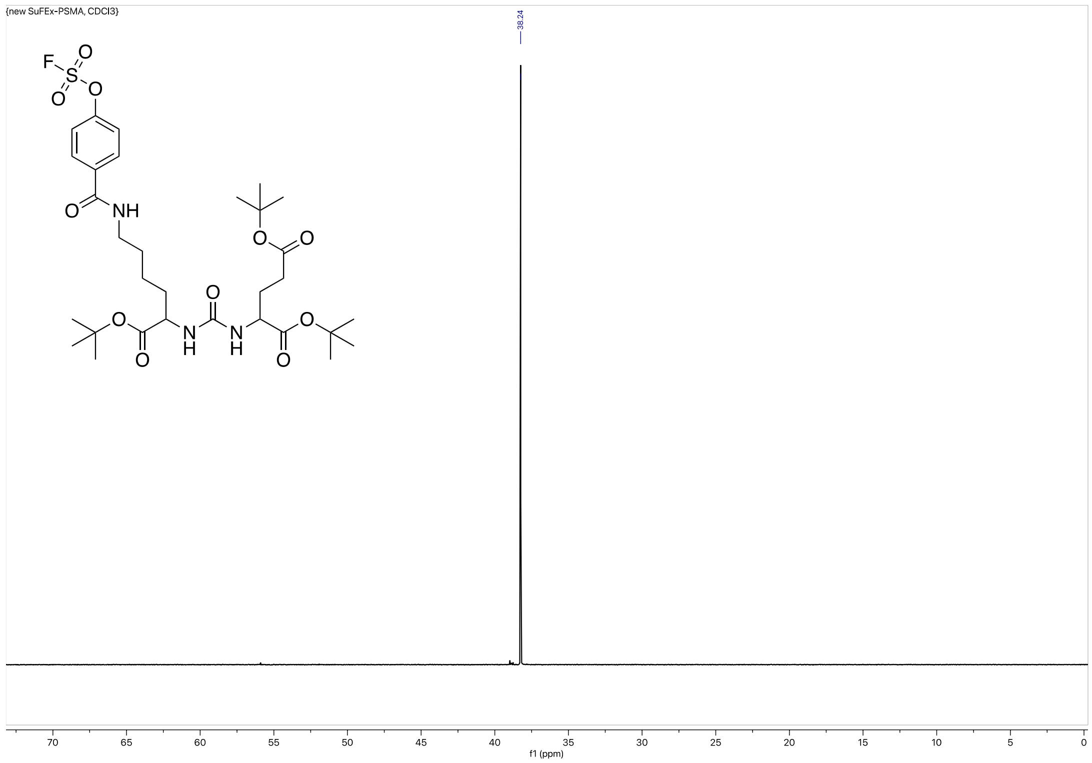

Figure 5. ^{19}F-NMR of di-tert-butyl ((1-(tert-butoxy)-6-(4-((fluorosulfonyl)oxy)benzamido)-1-oxohexan-2-yl)carbamoyl)glutamate (**4**).

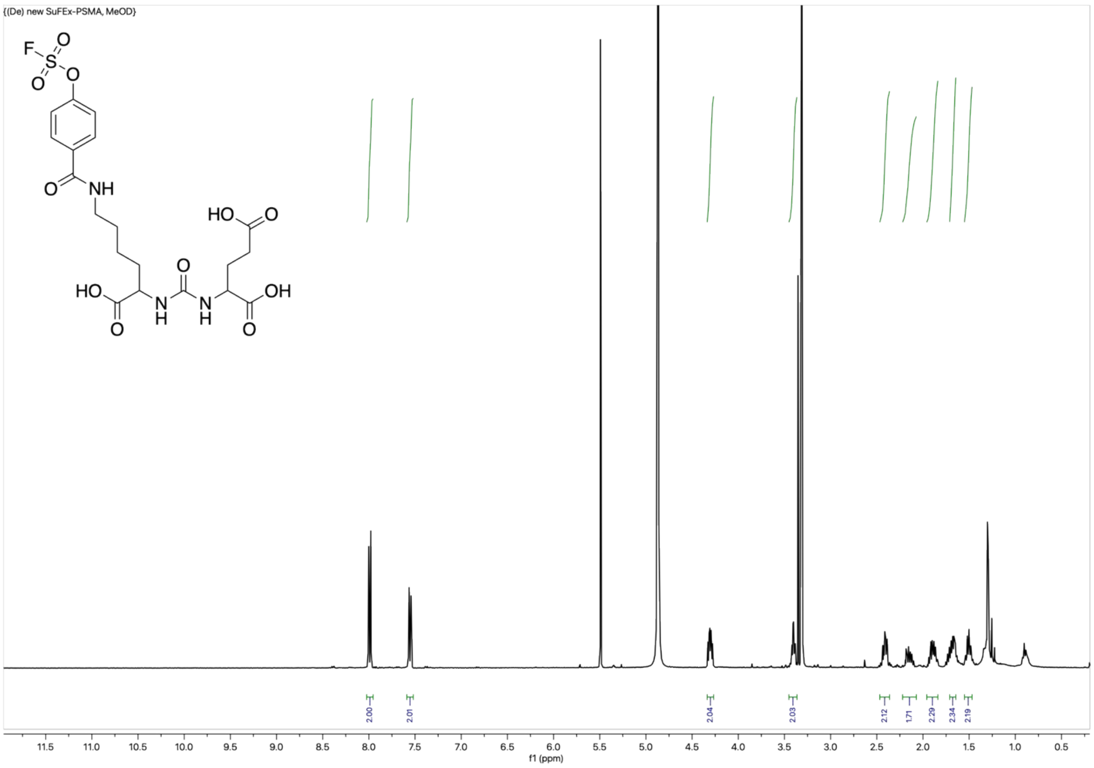

Figure 6. ^{1}H-NMR of ((1-carboxy-5-(4-((fluorosulfonyl)oxy)benzamido)pentyl)carbamoyl)glutamic acid (**5**).

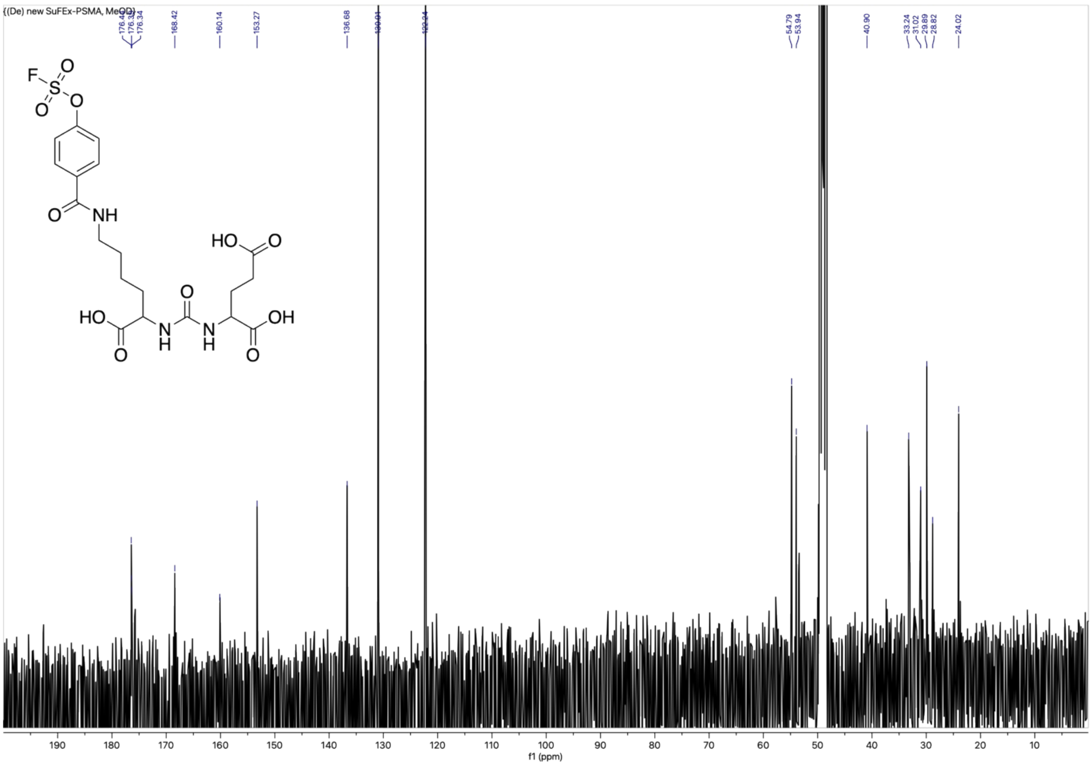

Figure 7. ^{13}C-NMR of ((1-carboxy-5-(4-((fluorosulfonyl)oxy)benzamido)pentyl)carb-amoyl)glutamic acid (**5**).

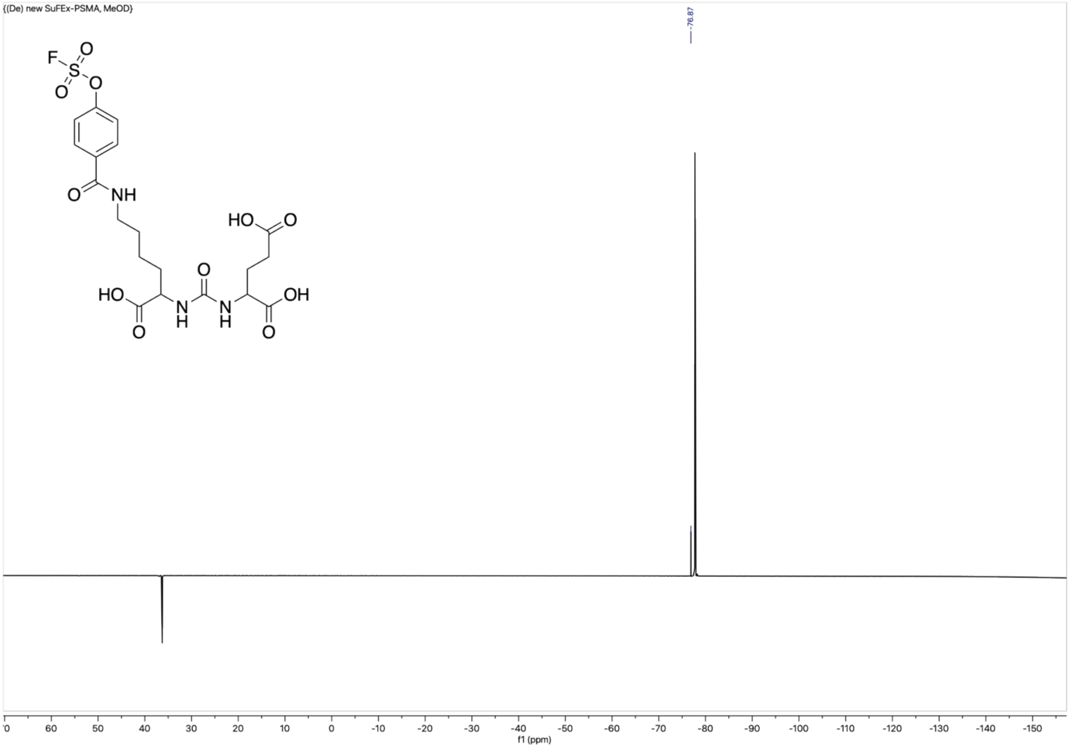

Figure 8. ^{19}F-NMR of ((1-carboxy-5-(4-((fluorosulfonyl)oxy)benzamido)pentyl)carb-amoyl)glutamic acid (**5**).

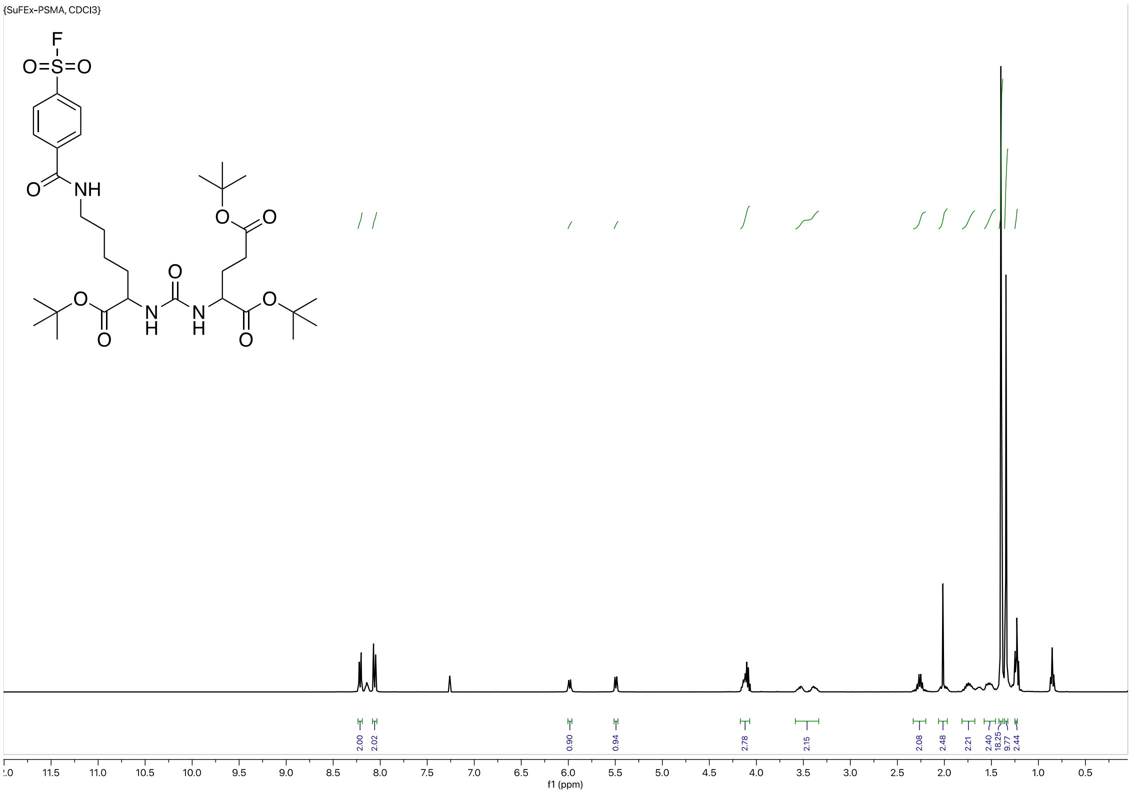

Figure 9. ^{1}H-NMR of di-tert-butyl ((1-(tert-butoxy)-6-(4-(fluorosulfonyl)benzamido)-1-oxohexan-2-yl)carbamoyl)glutamate (**6**).

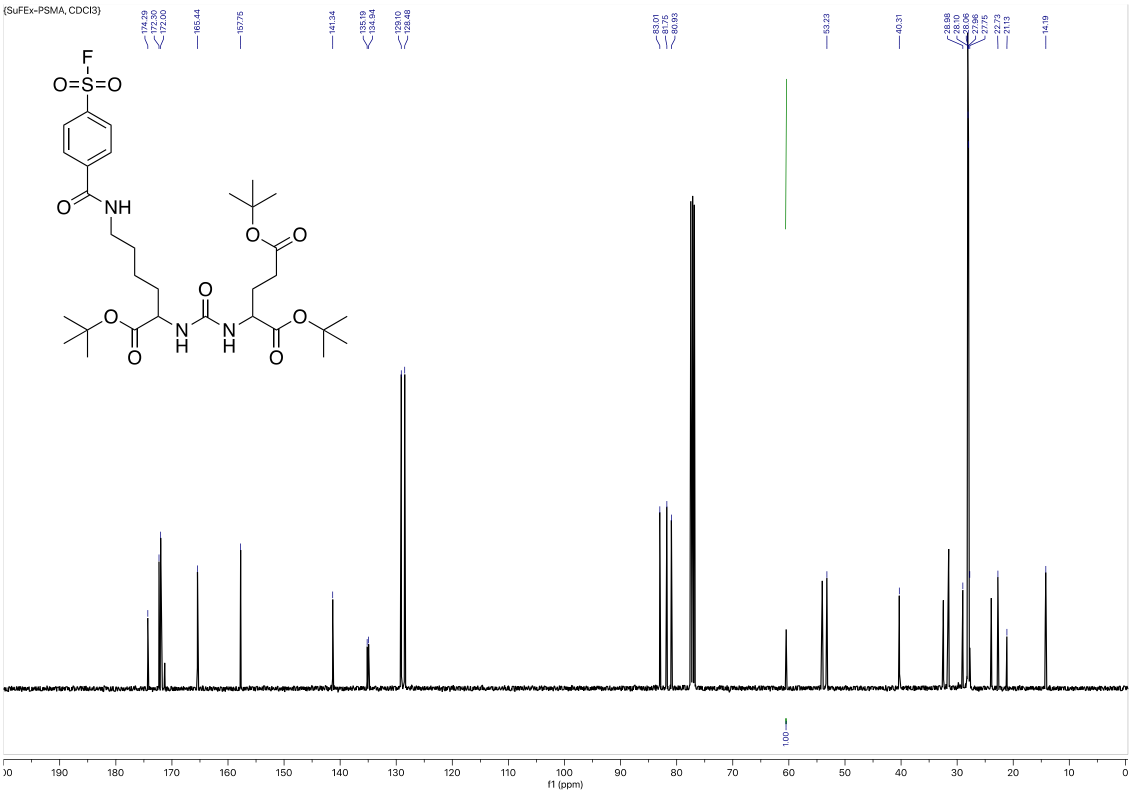

Figure 10. ^{13}C-NMR of di-tert-butyl ((1-(tert-butoxy)-6-(4-(fluorosulfonyl)benzamido)-1-oxohexan-2-yl)carbamoyl)glutamate (**6**).

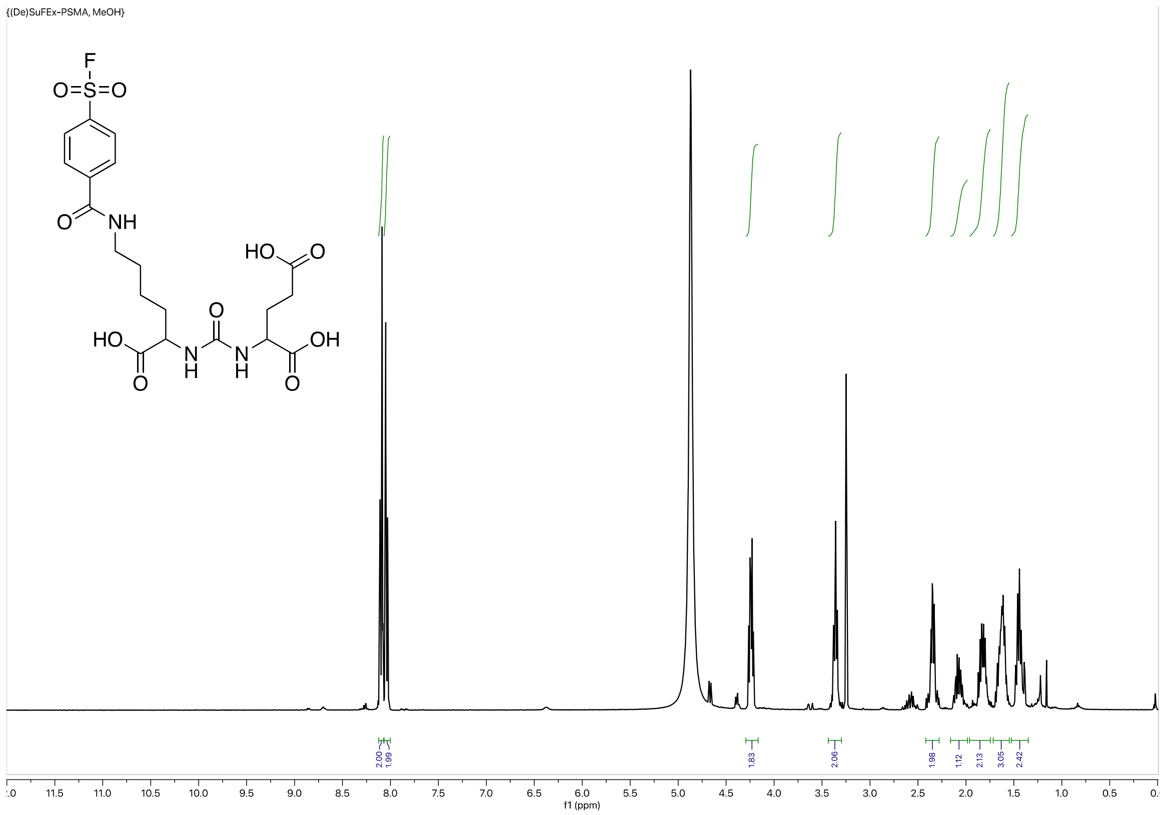

Figure 11. ^{1}H-NMR of ((1-carboxy-5-(4-((fluorosulfonyl)benzamido)pentyl)carbamoyl)glutamic acid (**7**).

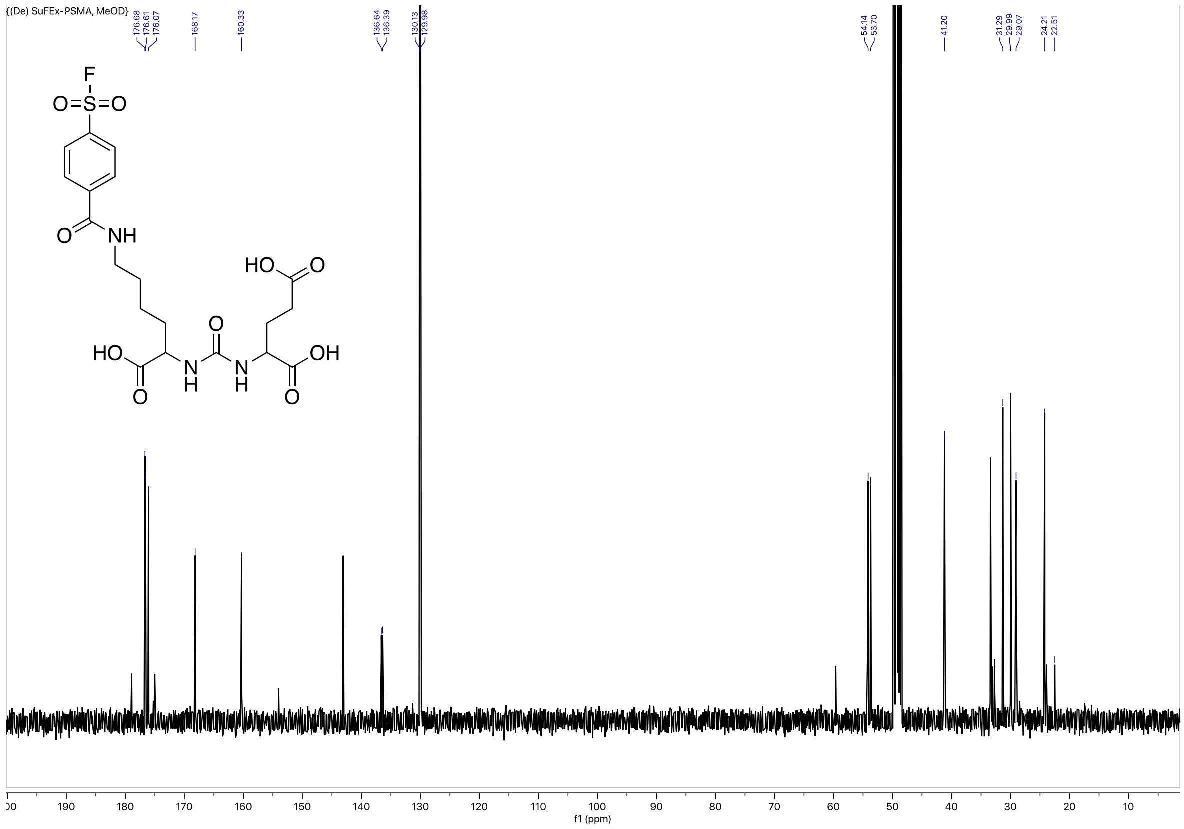

Figure 12. ^{13}C-NMR of ((1-carboxy-5-(4-((fluorosulfonyl)benzamido)pentyl)carbamoyl)glutamic acid (**7**).

2.4 Mass spectra

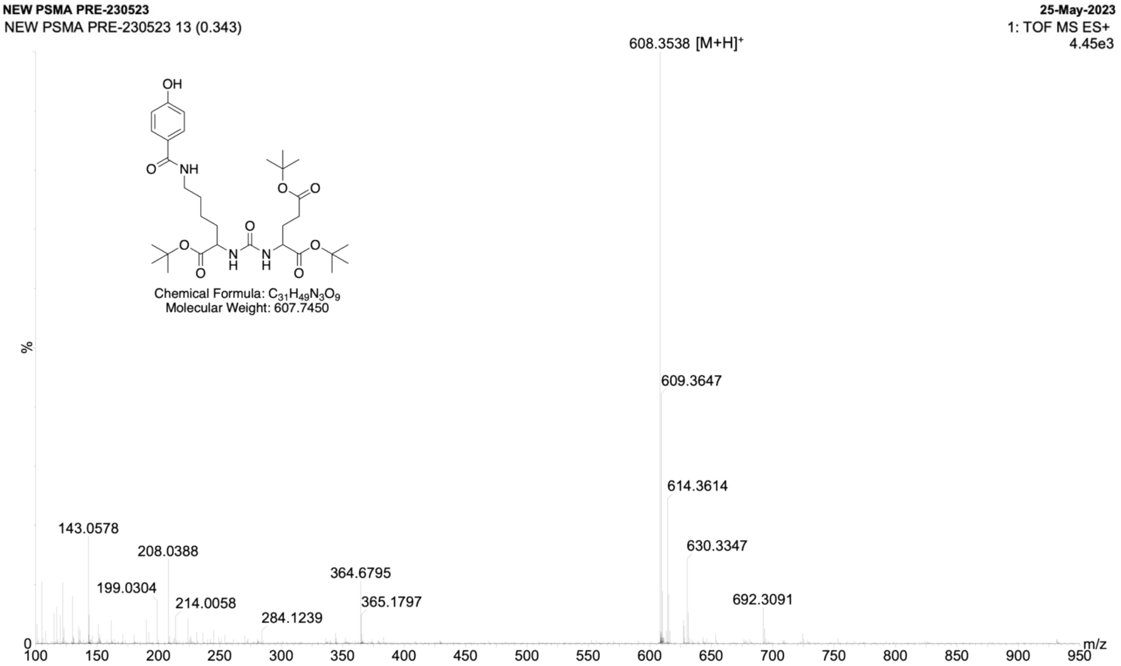

Figure 13. ES-MS of di-tert-butyl ((1-(tert-butoxy)-6-(4-hydroxybenzamido)-1-oxohexan-2-yl)carbamoyl)glutamate (**3**).

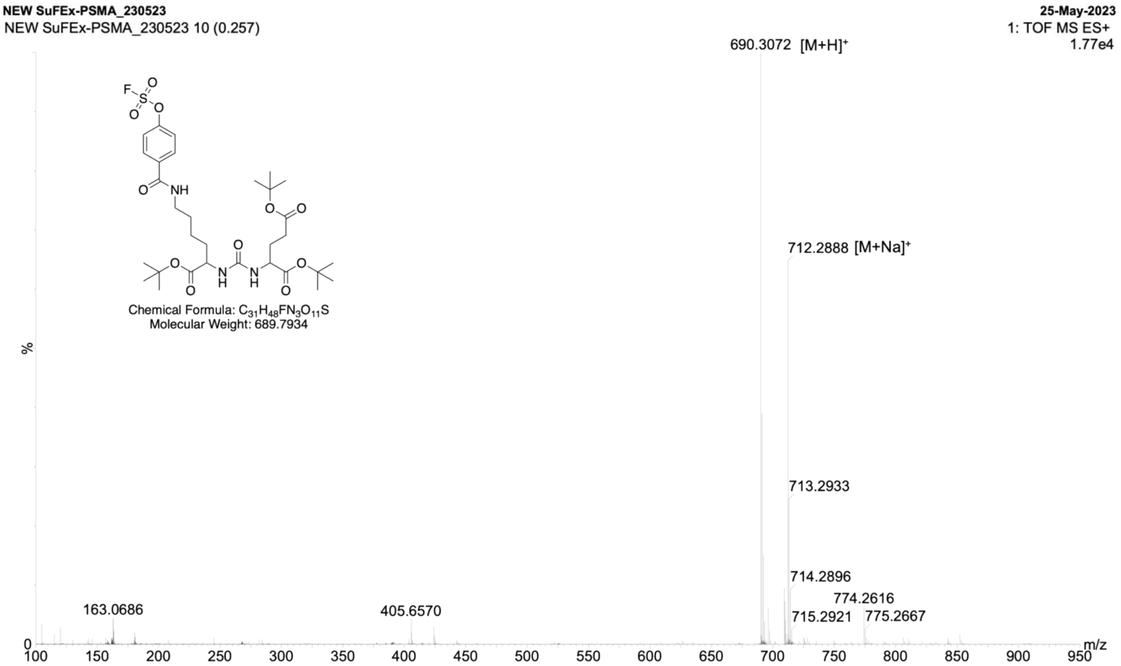

Figure 14. ES-MS of di-tert-butyl ((1-(tert-butoxy)-6-(4-((fluorosulfonyl)oxy)benzamido)-1-oxohexan-2-yl)carbamoyl)glutamate (**4**).

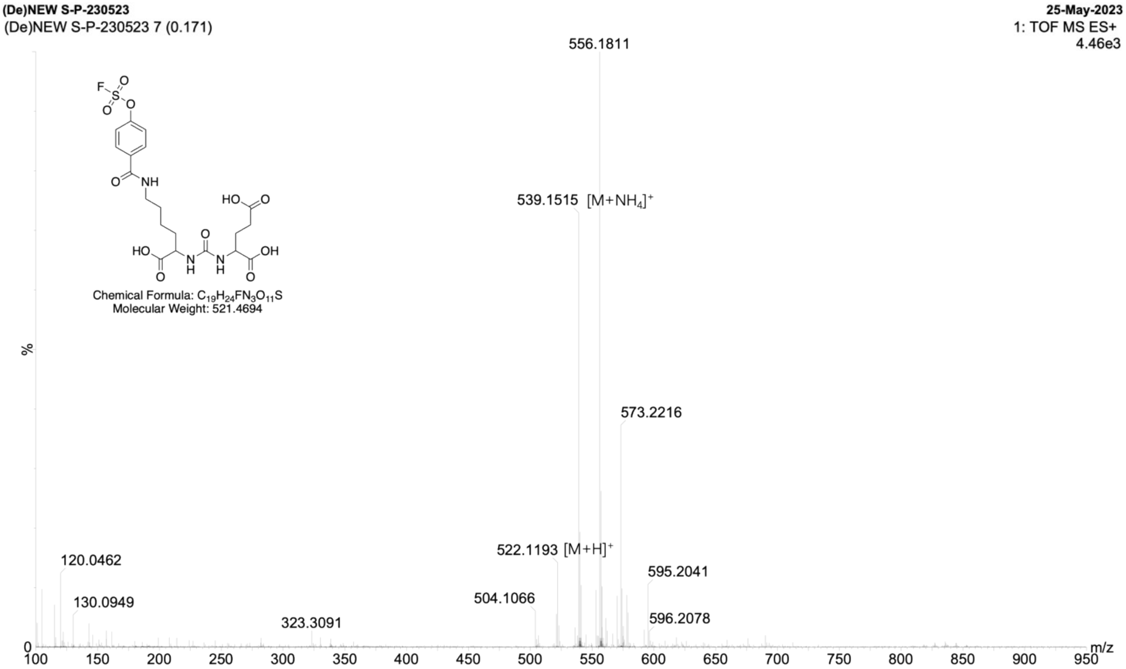

Figure 15. ES-MS of ((1-carboxy-5-(4-((fluorosulfonyl)oxy)benzamido)pentyl)carbamoyl)glutamic acid (**5**).

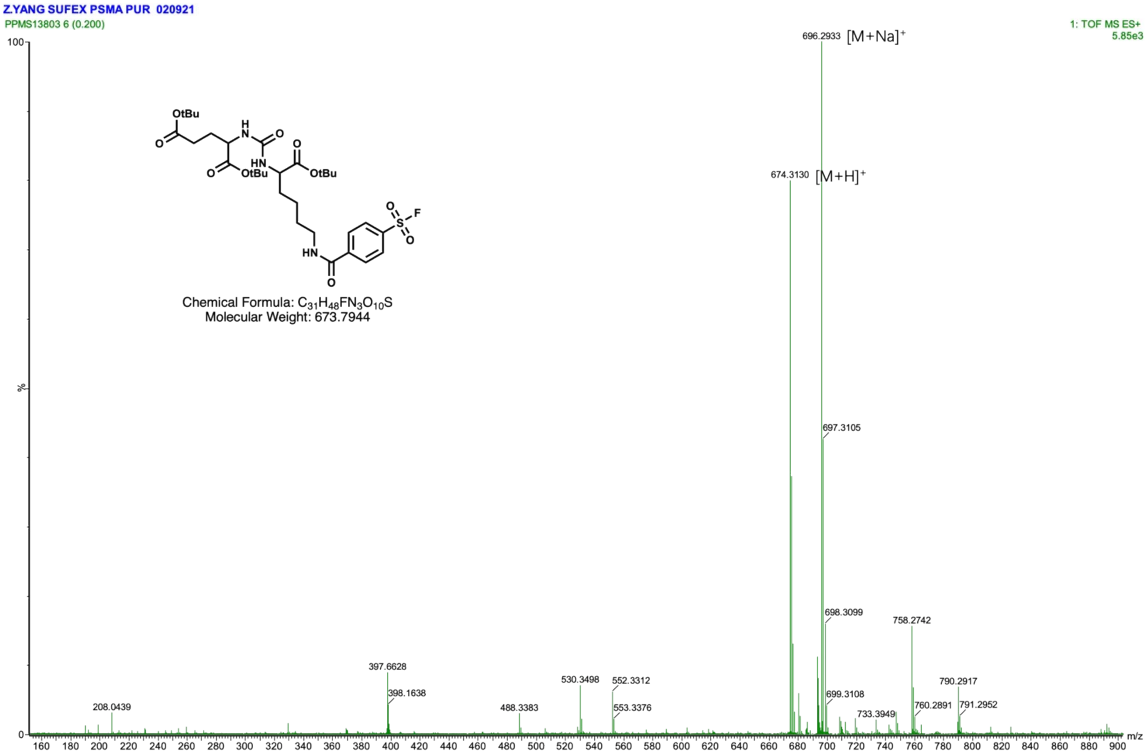

Figure 16. ES-MS of di-tert-butyl ((1-(tert-butoxy)-6-(4-(fluorosulfonyl)benzamido)-1-oxohexan-2-yl)carbamoyl)glutamate (**6**).

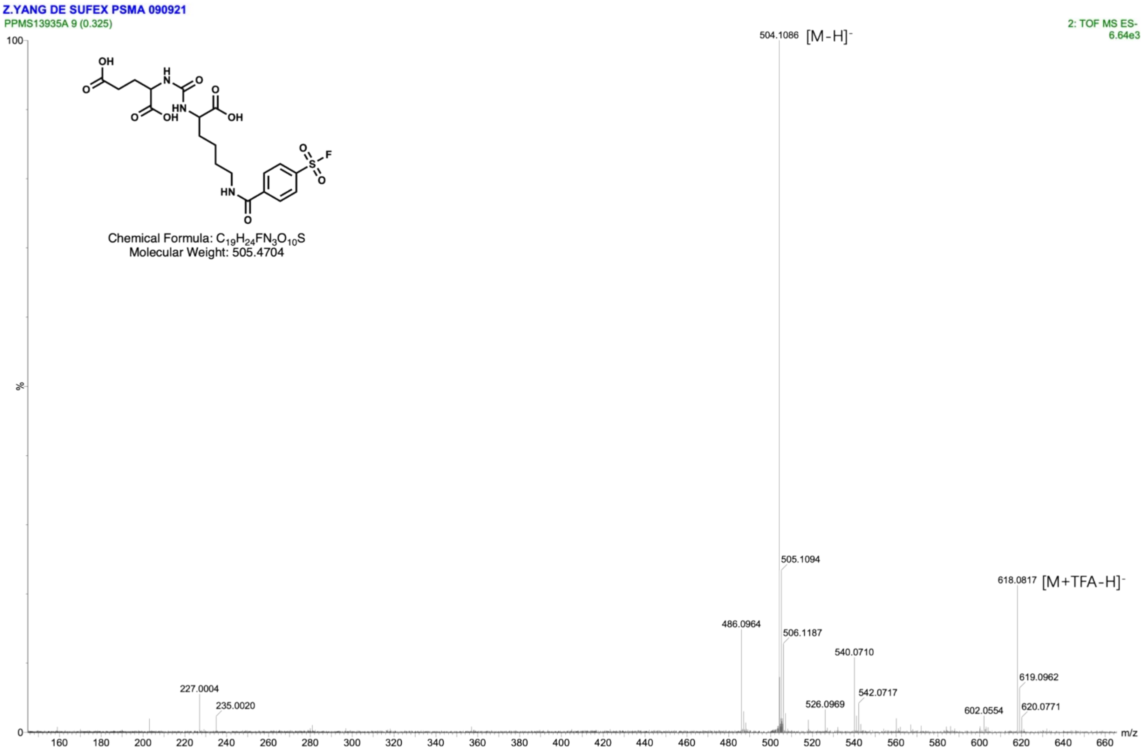

Figure 17. ES-MS of ((1-carboxy-5-(4-((fluorosulfonyl)benzamido)pentyl)carbamoyl)glutamic acid (**7**).

3.0 Radiochemistry

3.1 Automated method. No-carrier-added aqueous [^{18}F]fluoride in enriched ^{18}O water was delivered to the FASTlab™ radiosynthesis module and trapped on a Waters QMA-carbonate Sep-Pak SPE cartridge. The [^{18}F]fluoride was eluted into the reactor vial using 600 µL of eluent (6mg/mL Kryptofix 2.2.2 in 800 µL MeCN, 3.5 mg/mL K_2CO_3 in 200 µL H_2O) by syringe. The [^{18}F]fluoride was dried under nitrogen and vacuum at 120 °C for 6 min. Compound **3** (50 µL, 5 µmol) was then dissolved in anhydrous MeCN (1 mL) was added to the reaction vessel and allowed to react at room temperature for 5 min. HCl (1 mL, 4M) was added to the reaction vessel containing **[^{18}F]3** and heated at 60 °C for 15 min. After cooling, the crude was diluted to 10 mL with 0.1% H_3PO_4 solution following a reaction vessel rinse before purification by semi-preparative HPLC (Shimadzu LC20-AT pump attached to a custom-built system, equipped with a Phenomenex Jupiter C12, 4µ (250 × 10 mm) column). The mobile phase was 70% H_2O/30% MeCN+0.1% H_3PO_4 at purification was performed at flow rate of 3 mL/min. **[^{18}F]5** was collected as a fraction and diluted with 0.1% H_3PO_4 (45 mL) for concentration and reformulation by tC18 plus SPE cartridge. The cartridge was washed with water and dried under a flow of nitrogen and eluted with EtOH (1 mL) for dilution into PBS (<10% EtOH v/v) for biological evaluation. The tubing used in this method was supplied in the FASTLab Developer Kit (14 cm and 42 cm). Short tubing was used to connect SPE cartridges, whereas longer tubing was used to connect to external consumables and equipment (dilution vials and HPLC loop). A tutorial account describing best practices for automating ^{18}F-radiochemistry is available and may assist in the implementation and adaptation of this method.[1]

Table 1. Populated positions on the FASTLab™ cassette.

Pos.	Description
1	$H_2[^{18}O]O$ Recovery Vial
2	QMA Eluent<br>(1 mL, Kryptofix 2.2.2 [6 mg/mL) in 0.8 mL MeCN & K_2CO_3 [3.5 mg/mL in 0.2 mL H_2O)
3	1 mL syringe
4	QMA SPE (Waters QMA-carbonate Sep-Pak, 130 mg)
5	QMA Line
6	Activity Inlet
7	Reactor
8	Reactor
9	Dilution vial (50 mL) for semi-preparative HPLC (contains 7.5 mL, H_2O + 0.1% H_3PO_4 of diluent for loading onto the injection loop of a semi-preparative HPLC system). Vial vented, 16mm, 25-gauge needle.
10	Semi-preparative HPLC Injection Loop (10 mL)
11	5 mL syringe
12	Precursor (7.5 µmol, 1.4 mL anhydrous MeCN)
13	Hydrochloric acid (4M, 4 mL)
14	Empty
15	Water Bag Spike + Water Bag
16	Ethanol (4 mL)
17	Return cut vial (100 mL) from HPLC (contains 40 mL H_2O + 0.1% H_3PO_4 of diluent for reformulation by tC18 SPE into a biocompatible milieu). Vial vented, 16mm, 25-gauge needle.
18	Line to C18 SPE
19	tC18 Plus
20	Final product out into vial containing PBS (9 mL).
21	Empty
22	Empty
23	Empty
24	5 mL syringe
25	Reactor

3.2 Manual method. No-carrier-added aqueous [^{18}F]fluoride in enriched ^{18}O water was delivered trapped on a QMA cartridge and released into a reaction vessel with 600 µL of eluent (2.4 mg Kryptofix 2.2.2 in 500 µL MeCN, 2.6 mg/mL $KHCO_3$ in 100 µL H_2O). The K_{222}-K[^{18}F]F complex was azeotropically dried by the addition of MeCN (3 × 0.7 mL) at 100 ºC under a gentle stream of inert gas. Precursor **4** (0.5 mg) was dissolved in 0.5 mL of MeCN and added to the dry K_{222}-K[^{18}F]F complex and the reaction was stirred at RT for 20 min. The reaction mixture was passed through an activated silica cartridge and flushed with dry MeCN (500 µL). The solvent volume was reduced under a gentle stream of inert gas at 60 ºC to around 200 µL, to which HCl (200 µL of 4 M) was added to the reaction mixture and stirred for 20 min at 60 ºC. The reaction mixture was neutralized by the addition of sodium acetate (3 M, pH 4) and purified by HPLC (Merck Discovery 250 × 10 mm C18 column eluted with a gradient 40 to 60% of MeOH + 0.1% TFA / H_2O + 0.1% TFA, 4.7 mL/min. The collected HPLC fraction was diluted with water (15 mL) and reformulated using a tC18 (145 mg) SPE cartridge preconditioned with EtOH (1 mL) and saline (5 mL). Trapped product was washed with saline (5 mL) and eluted in EtOH (200 µL) followed by sodium acetate (0.1 N, pH 6.7).

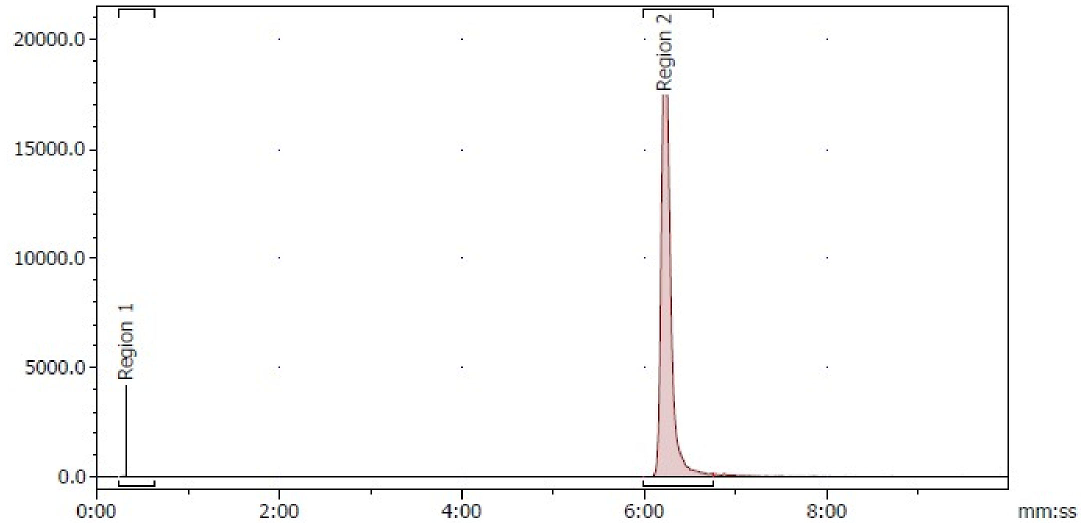

Figure 18. Representative radio-HPLC chromatogram of [^{18}F]SO_3F-PSMA (**[^{18}F]5**). R_f = 6:13 (mm:ss), 99.7% ROI.

4.0 Biological Evaluation

4.1 Cell culture

C4-2B, LNCaP, PC3, and PNT1A cell lines (obtained from Prof Charlotte Bevan, Imperial College London) were grown in RPMI medium (Sigma Aldrich, Gillingham, UK). The media was supplemented with 10% fetal calf serum, 1% L-glutamine and 2% penicillin–streptomycin (Life Technologies). All cells were cultured at 37 °C in a humidified atmosphere containing 5% CO_2. All cells were routinely tested for mycoplasma and typically not passaged for longer than three months.

4.2 Flow Cytometry

To analyse cell surface markers, 1.5 x10^5 cells were collected and centrifuged at 1500 rpm for 5 min. The cell pellet was then resuspended in PBS, followed by the addition of Alexa Fluor® 488 anti-human PSMA (FOLH1) Antibody (342505, BioLegend) at the concentration indicated by the manufacturer. After 30 min incubation at 4 °C, cells were washed and suspended in PBS for analysis. Live/Dead fixable Near-IR dead cell stain kit (L34976, Invitrogen) was used to determine cell viability by flow cytometry. 30,000 events per sample were acquired on FACS Canto flow cytometer (Becton Dickinson Immunocytometry Systems) with FACS Diva Software version 4.0.2. Data obtained were analysed using FlowJo software v7.6 (FlowJo, LLC). Unstained and isotype controls were used to define gates.

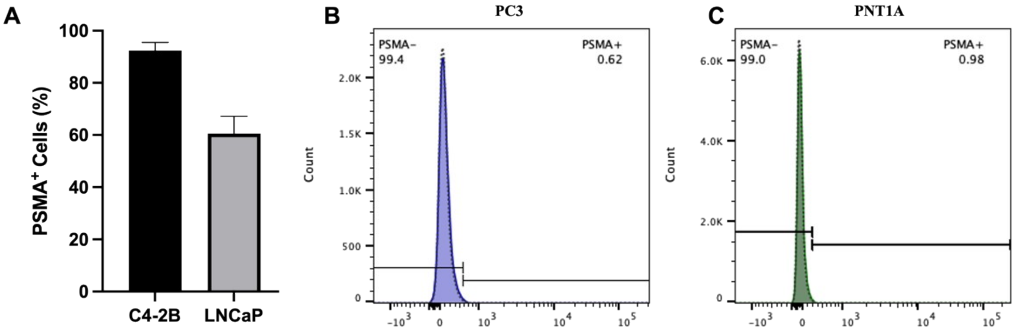

Figure 19. Prostate cancer cell line validation. **A)** Cell surface expression of PSMA in C4-2B and LNCaP cells. Data from two independent experiments presented as mean ± SEM; Representative histograms of PSMA expression in **B)** PC3 and **C)** PNT1A cells; two independent experiments performed, with the dotted black line representing cells stained with isotype control antibody (n=3).

4.3 *In vitro* cell uptake of [^{18}F]SO_3F-PSMA ([^{18}F]5)

PSMA positive cell lines: C4-2B, LNCaP and PSMA negative cell lines: PC3, PNT1A were plated at a seeding density of 5× 10^5 cells per well in 6-well plates. After 24 h, cells were incubated with 22.2 MBq of **[^{18}F]5** for 1 h at 37 °C and 5% CO2. Cells were then washed three times with ice-cold PBS and lysed in RIPA buffer (0.5 mL per well) for 15 min on ice. Cell-bound radioactivity was measured, and decay corrected using Packard Cobra II gamma counter (Perkin Elmer). The radiopharmaceutical uptake was normalised to total cellular protein as measured by the BCA assay. Uptake was expressed as % radioactivity/mg protein. All experiments were carried out in quadruplicate or more (n = 4-6).

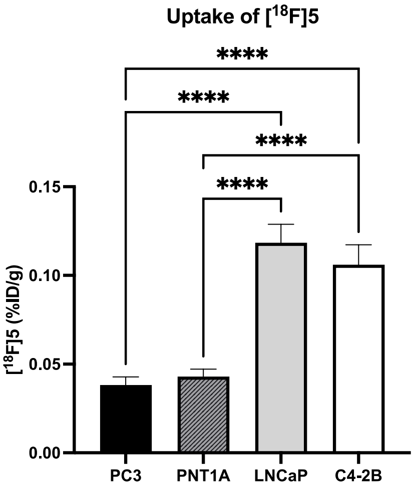

Figure 20. *In vitro* uptake of **[^{18}F]5** in a panel of cell lines with differential PSMA expression.

4.4 *In vivo PET imaging of* cell [^{18}F]SO_3F-PSMA ([^{18}F]5) in tumour-naïve mice

BALB/c mice (n=4) were anesthetized with 2% isoflurane/O_2 anaesthesia and scanned using Siemens Inveon small-animal multimodality PET/CT system (Siemens Healthcare Molecular Imaging). Following the completion of the CT scan, mice were injected with **[^{18}F]5** (3.605 ± 0.647 MBq) intravenously via the lateral tail vein. Dynamic emission scans were acquired in list-mode format over 0–60 minutes to give decay-corrected values of radioactivity accumulation in tissues. Image data were sorted into 0.5 mm sinogram bins, and 33 timeframes and images were reconstructed using 2D-ordered subsets expectation

maximization (2D-OSEM) algorithm with CT-based attenuation correction. Cumulative images of the data were used for visualization of radiotracer uptake using Siemens Inveon Research Workplace software (Siemens Molecular Imaging, Inc. Knoxville, USA). PET and CT images were co-registered and used to draw three-dimensional regions of interest (ROIs) over tissues to obtain time-activity curves (TACs).

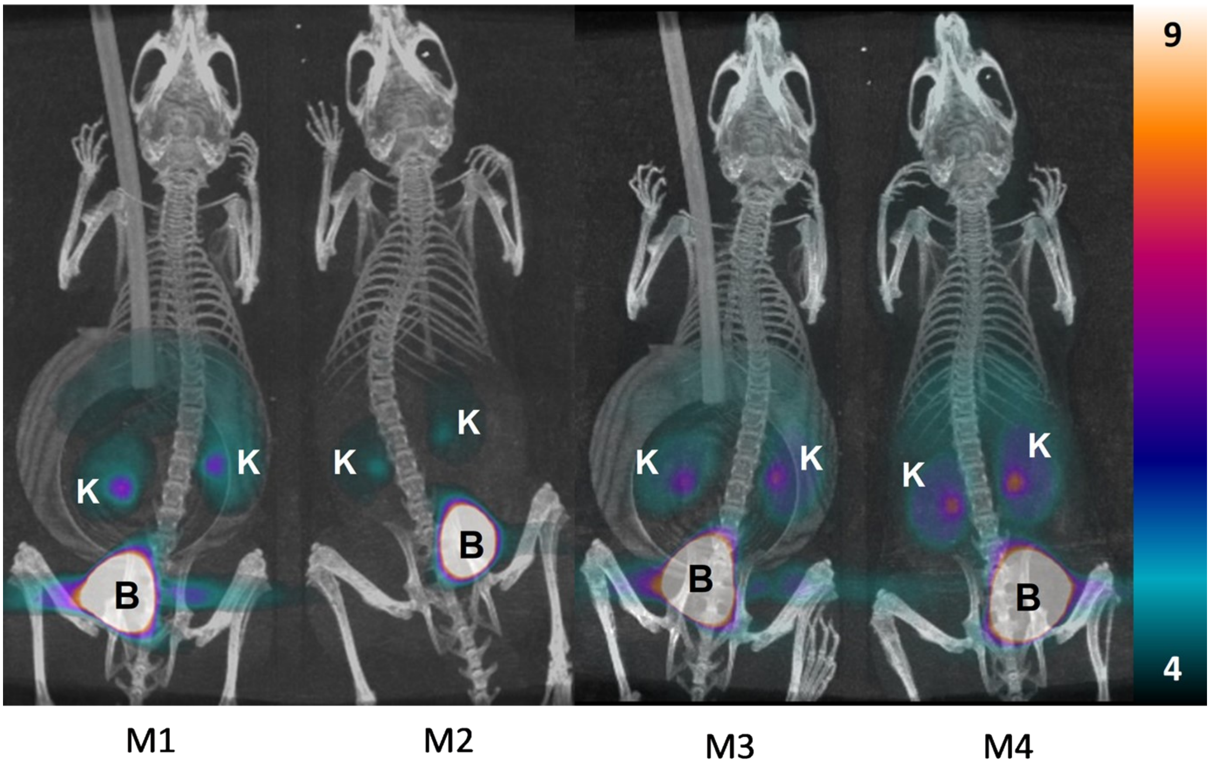

Figure 21. Dynamic PET/CT image of BALB/c mice. K = kidney, B = bladder.

4.5 Metabolite analysis of [^{18}F]SO$_3$F-PSMA ([^{18}F]5)

Liver, urine, blood plasma and kidney were analysed for radioactive metabolites by radio-HPLC (Agilent 1100 system) fitted with an in-line posiRAM metabolite detector (LabLogic, Sheffield, UK). A solvent system of A: H2O + 0.1% TFA and B: MeCN + 0.1% TFA was used following a gradient of A: 95%, B: 5% (0 - 5 min), A: 5%, B: 95% (5 - 16 min), A: 95%, B: 5% (16 – 18 min), A: 95%, B: 5% (18 – 20 min). The liver was excised, and a portion homogenised in ice cold MeCN (1 mL) using a Precellys tissue homogeniser (Stretton Scientific Ltd, Derbyshire, UK). Solid tissues and proteins were pelleted by centrifugation (13,000 g, 5 min) and the supernatant was removed and filtered (0.22 µm syringe filter) before being diluted in H_2O + 0.1% TFA for radio-HPLC analysis. The same processes were followed for kidney. Plasma was obtained from whole blood by centrifugation (2000 g, 5 min) to separate the blood cells from plasma. The plasma was removed, and proteins precipitated in ice cold MeCN (1 mL) and centrifuged (13,000 g, 5 min) to pellet the proteins. The supernatant was filtered (0.22 µm syringe filter) and diluted in H_2O + 0.1% TFA for radio-HPLC analysis. Urine was diluted in H_2O + 0.1% TFA and injected after filtration (0.22 µm syringe filter). The injection loop (100 µL) was washed with mobile phase between each injection. Radio-HPLC chromatograms were integrated using Laura 6 software (LabLogic, Sheffield, UK).

4.6 Animal tumour model

Male BALB/c nude mice (6 – 8 weeks old) were implanted subcutaneously with LNCaP cells at 7.5 x10^6 on the right shoulder, for use with dynamic PET imaging. Inoculations were performed under anaesthesia (2% isoflurane/O_2). All mice were 12 weeks of age with similar weights (23.0 ± 2.4 g) and tumour volumes (212 ± 10 mm^3) and kept under standard conditions in individually ventilated cages with animal food provided *ad libitum* prior to experiments.

4.7 *In vivo PET imaging of* cell [^{18}F]SO_3F-PSMA ([^{18}F]5) in LNCaP tumour bearing mice

Male BALB/c mice (Janvier Laboratories) bearing LNCaP tumours were anesthetised and scanned on a dedicated small animal PET scanner (Molecubes, Ghent, Belgium) following a bolus injection (200 μL) of 0.90 ± 0.09 MBq of **[^{18}F]5** *via* a lateral tail vein cannula (n = 3). Imaging was performed under anaesthesia (2% isoflurane/O_2) and scans were acquired over 0-90 min (dynamic) to give decay-corrected values of radioactivity accumulation in tissues. Data were reconstructed using an Ordered Subset Expectation Maximisation (OSEM) iterative reconstruction algorithm, with 30 iterations, an intrinsic voxel size of 400 μm and attenuation correction from a CT generated attenuation map. Cumulative images of the data were used for visualisation of radioactivity uptake and average data were used for quantification by defining regions of interests (ROIs) in VivoQuant (Invicro, London, UK) to generate time activity curves.

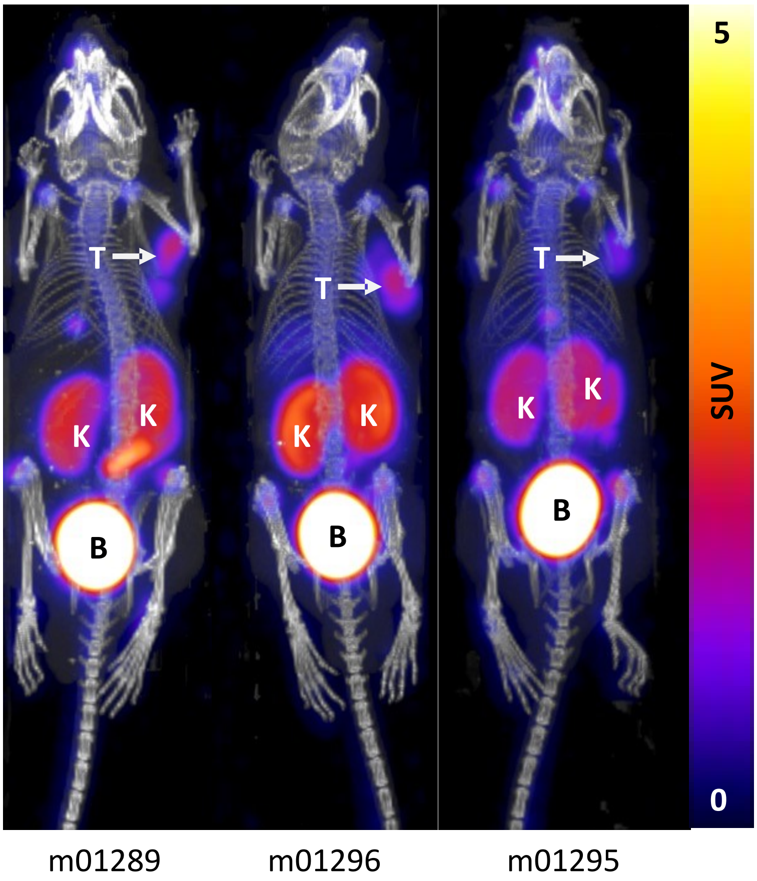

Figure 22. Dynamic PET/CT image of male BALB/c nude mice bearing LNCaP tumours. Frames summed from 80 – 90 min. K = kidney, B = bladder, T = tumour.

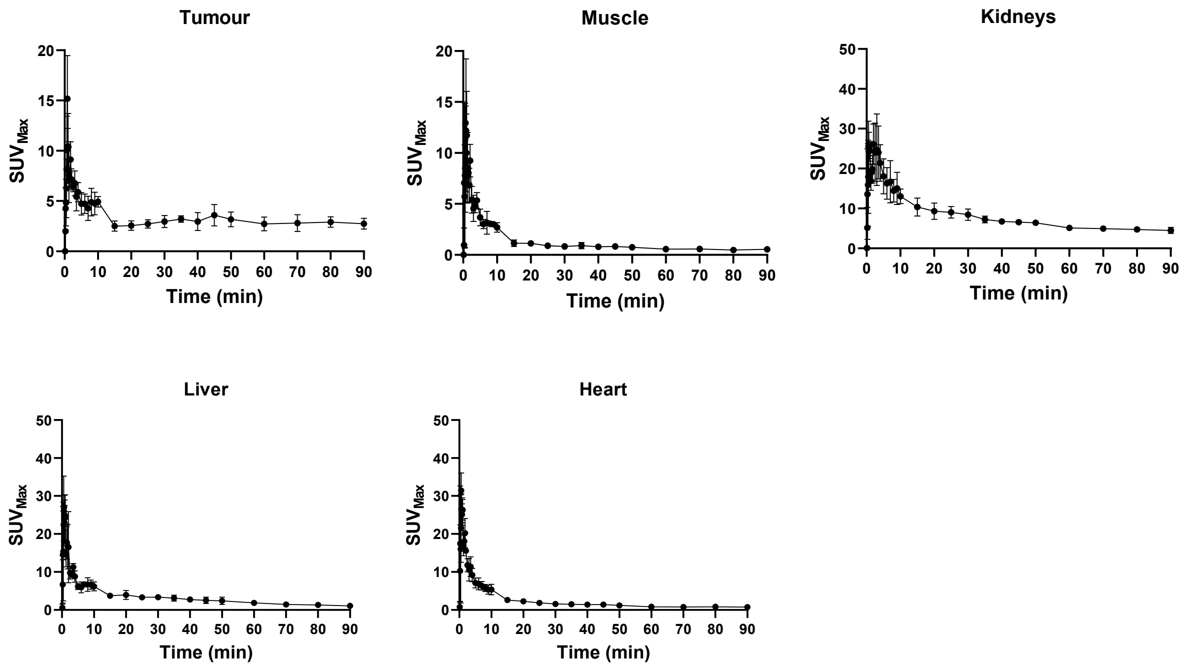

Figure 23. Time activity curves showing uptake (SUV_{Max}) over a 90 min dynamic PET scan.

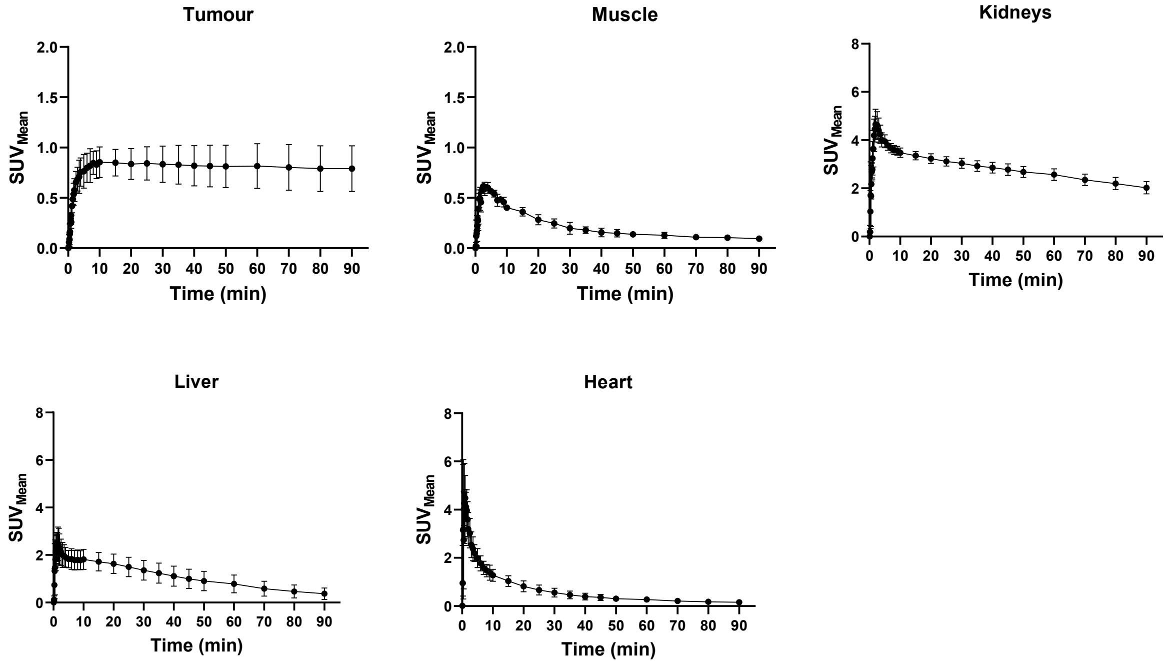

Figure 24. Time activity curves showing uptake (SUV_{Mean}) over a 90 min dynamic PET scan.

4.8 *In vitro* cell uptake of $[^{18}F]SO_2F$-PSMA ($[^{18}F]$7)

PSMA positive cell lines: C4-2B, LNCaP, and PSMA negative cell lines: PC3, PNT1A were plated at a seeding density of 5× 10^5 cells per well in 6-well plates. After 24 h, cells were incubated with 22.2 MBq of **[^{18}F]7** for 1 h at 37 °C and 5% CO2. Cells were then washed three times with ice-cold PBS and lysed in RIPA buffer (0.5 mL per well) for 15 min on ice. Cell-bound radioactivity was measured, and decay corrected using Packard Cobra II gamma counter (Perkin Elmer). The radiopharmaceutical uptake was normalised to total cellular protein as measured by the BCA assay. Uptake was expressed as % radioactivity/mg protein. All experiments were carried out in quadruplicate or more (n = 4-6).

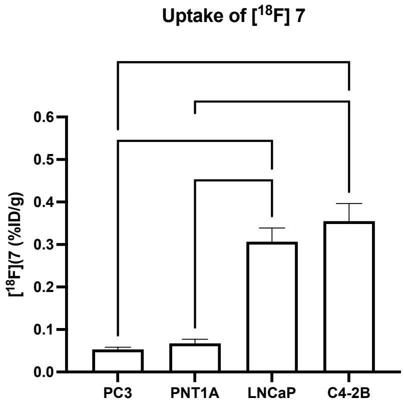

Figure 25. In vitro cell uptake of **[^{18}F]7** in a panel of cell lines with differential expression of PSMA.

4.9 *In vivo PET imaging of* [^{18}F]SO_2F-PSMA ([^{18}F]7) in naïve mice

C57BL/6 mice (n=2) were anesthetized with 2% isoflurane/O_2 anaesthesia and scanned using Siemens Inveon small-animal multimodality PET/CT system (Siemens Healthcare Molecular Imaging). Following the completion of the CT scan, mice were injected with **[^{18}F]7** (2.325 ± 0.175 MBq) intravenously via the lateral tail vein. Dynamic emission scans were acquired in list-mode format over 0–60 minutes to give decay-corrected values of radioactivity accumulation in tissues. Image data were sorted into 0.5 mm sinogram bins, and 33 timeframes and images were reconstructed using 2D-ordered subsets expectation maximization (2D-OSEM) algorithm with CT-based attenuation correction. Cumulative images of the data were used for visualization of radiotracer uptake using Siemens Inveon Research Workplace software (Siemens Molecular Imaging, Inc. Knoxville, USA). PET and CT images were co-registered and used to draw three-dimensional regions of interest (ROIs) over tissues to obtain time-activity curves (TACs).

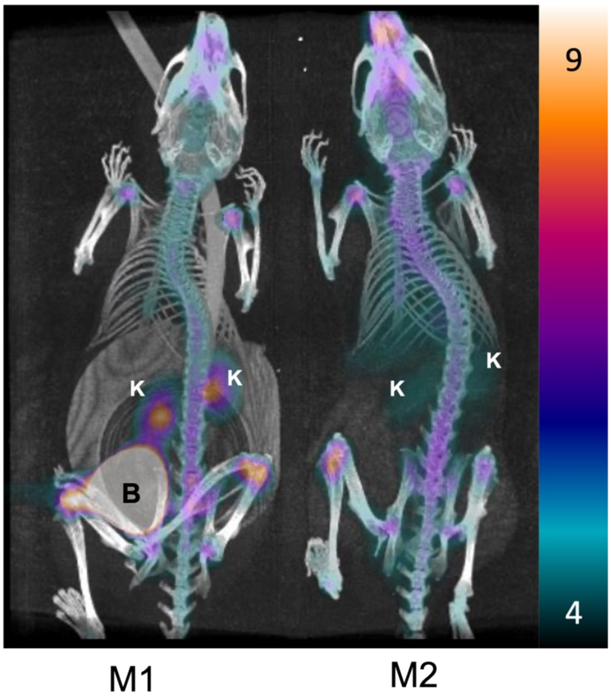

Figure 26. Dynamic PET/CT image of C57BL/6 mice. K = kidney, B = bladder. (M2 Bladder not filled, no urine)

4.10 Metabolite analysis of [^{18}F]SO$_2$F-PSMA ([^{18}F]7)

The protocol for radioactive metabolite analysis outlined in ESI Section 4.5 was followed. Radioactivity was only detected in plasma and urine, as shown in figure 22.

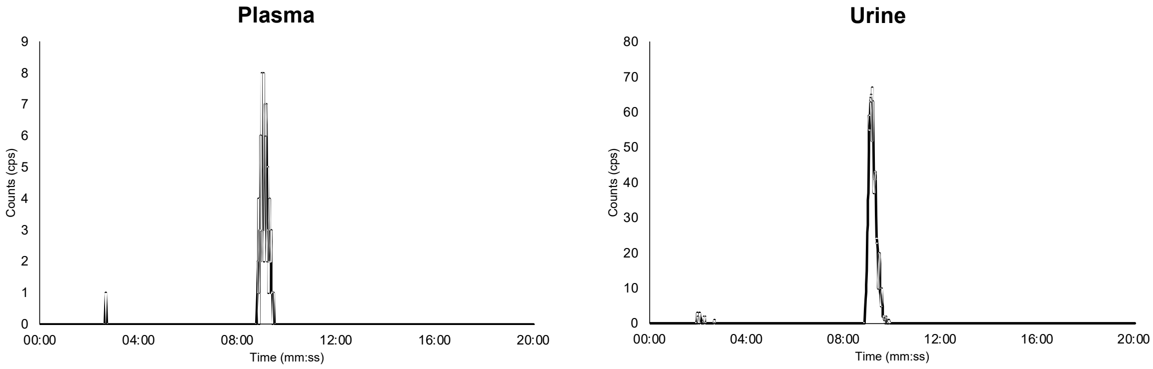

Figure 27. Radioactive metabolite analysis HPLC chromatograms at 60 min p.i. of [^{18}**F]7** showing polar metabolites, likely to be free [^{18}F]fluoride.

4.11 Ethics Statement

All animal experiments were done by licensed investigators in accordance with the UK Home Office Guidance on the Operation of the Animal (Scientific Procedures) Act 1986 (HMSO, London, UK, 1990) and within guidelines set out by the UK National Cancer Research Institute Committee on Welfare of Animals in Cancer Research.

References

1. C. Barnes, M. Nair, E. O. Aboagye, S. J. Archibald and L. Allott, Reaction Chemistry & Engineering, 2022, 7, 2265-2279.
